# Use of dietary indices to control for diet in human gut microbiota studies

**DOI:** 10.1186/s40168-018-0455-y

**Published:** 2018-04-25

**Authors:** Ruth C. E. Bowyer, Matthew A. Jackson, Tess Pallister, Jane Skinner, Tim D. Spector, Ailsa A. Welch, Claire J. Steves

**Affiliations:** 1 The Department of Twin Research, Kings College London, 3-4th Floor South Wing Block D, St Thomas’ Hospital, Westminster Bridge Road, London, SE1 7EH UK; 2 Section of Nutrition and Metabolism, International Agency for Research on Cancer (IARC-WHO), 150 Cours Albert Thomas, 69008 Lyon, France; 3 Norwich Medical School 2.02, Faculty of Medicine and Health Sciences, University of East Anglia, Norwich, NR4 7TJ UK; 4 Clinical Age Research Unit, Kings College Hospital Foundation Trust, London, UK

**Keywords:** Microbiome, Microbiota, Dietary Index, Dietary covariate, Human microbiota, Food frequency questionnaire, FFQ, Healthy Eating Index, HEI, Mediterranean Dietary Score, MDS, Healthy Food Diversity Index, HFD-Index

## Abstract

**Background:**

Environmental factors have a large influence on the composition of the human gut microbiota. One of the most influential and well-studied is host diet. To assess and interpret the impact of non-dietary factors on the gut microbiota, we endeavoured to determine the most appropriate method to summarise community variation attributable to dietary effects. Dietary habits are multidimensional with internal correlations. This complexity can be simplified by using dietary indices that quantify dietary variance in a single measure and offer a means of controlling for diet in microbiota studies. However, to date, the applicability of different dietary indices to gut microbiota studies has not been assessed. Here, we use food frequency questionnaire (FFQ) data from members of the TwinsUK cohort to create three different dietary measures applicable in western-diet populations: The Healthy Eating Index (HEI), the Mediterranean Diet Score (MDS) and the Healthy Food Diversity index (HFD-Index). We validate and compare these three indices to determine which best summarises dietary influences on gut microbiota composition.

**Results:**

All three indices were independently validated using established measures of health, and all were significantly associated with microbiota measures; the HEI had the highest *t* values in models of alpha diversity measures, and had the highest number of associations with microbial taxa. Beta diversity analyses showed the HEI explained the greatest variance of microbiota composition. In paired tests between twins discordant for dietary index score, the HEI was associated with the greatest variation of taxa and twin dissimilarity.

**Conclusions:**

We find that the HEI explains the most variance in, and has the strongest association with, gut microbiota composition in a western (UK) population, suggesting that it may be the best summary measure to capture gut microbiota variance attributable to habitual diet in comparable populations.

**Electronic supplementary material:**

The online version of this article (10.1186/s40168-018-0455-y) contains supplementary material, which is available to authorized users.

## Background

The composition of the gut microbiota is associated with various aspects of human health and by many is considered a new clinical target [1]. Genetic influences are thought to be low, with environmental factors being the primary drivers of variation [2, 3]. Research has focused on host-mediated environmental factors such as xenobiotic exposure, antibiotic use and, in particular, diet, where multiple studies have indicated associations of long-term diet with the microbiota [4–6]. For example, non-digestible fermentable dietary carbohydrates, short-chain fatty acid ratios and dietary protein and fat can modulate bacterial abundance [7–11]. However, the extent to which clinical interventions or more distal factors, such as socio-economics and geo-physical factors influence the microbiota are emerging questions [12–14]. Selecting a dietary measure which encapsulates the variance in the microbiota attributable to diet is a useful goal which enables adjustment for diet in many studies. However, currently there is no standard approach to quantification of dietary data in microbiota studies.

Diet is a complex, multi-faceted phenotype that is often summarised using dietary indices to simplify analyses [15]. Dietary indices are nutritionally derived indices based on levels of (often differently defined) healthy consumption of nutrients or food groups. Analysing diet with the focus on patterns rather than individual dietary constituents is advantageous because dietary constituents are consumed together and often correlate with one another [16]. Dietary indices therefore provide a means to capture the overall dietary pattern of an individual or population in a single measure, allowing adequate adjustment for diet without saturation of models by the high dimensionality of dietary data. Dietary indices tend to assess diet quality based broadly on one of three categories; consumption measured against dietary guidelines, recommend foods, and dietary variety [17]. Indices within this analysis were selected to fall broadly into one of these three categories and because they were not defined in relation to a specific disease.

### Dietary indices

#### Healthy Eating Index (HEI)

The Healthy Eating Index (HEI) 2010 is a dietary index developed by the United States Department of Agriculture (USDA) as a means to assess diet measured as compliance to US Dietary Guidelines for Americans [15]. Designed to capture diet quality from 24-h food recalls and FFQ data, the HEI is comprised of 12 calorie-adjusted components representing ‘adequacy’ components, scored to reflect the extent an individual meets the recommended consumption level for that group, and ‘moderation’ components, where maximum scores are awarded when consumption falls below a lower threshold. The HEI is scored from 0 to 100; the higher an individual’s score therefore, the healthier their diet is considered to be. The HEI was selected for this analysis because it is readily applicable to FFQ data [18]; it contains relative weighted measures for each group; and because it uses set thresholds (i.e. rather than those based on study population averages).

#### Mediterranean Dietary Score (MDS)

Mediterranean diets are associated with lower rates of common chronic diseases. They are characterised by high intakes of whole grain, vegetables, legumes, fruit, unsaturated lipids and fish; low to medium intakes of saturated lipids, meat and dairy, and modest alcohol consumption [19]. The Mediterranean Dietary Score (MDS), scored from 0 to 10, is considered here as an index based on study population averages; due to its increasing popularity as a measure of dietary health [20]; and because of its straightforward method of grouping foods. Here, we use methodology developed and evaluated for use in non-Mediterranean countries [19].

#### Healthy Food Diversity index (HFD-index)

Indices that capture dietary diversity may offer researchers a fast and effective way of assessing dietary quality, based on suggestive evidence that a more diverse diet may be associated with better health outcomes [21]. In addition, we hypothesised that a wider variety of foods may result in a wider variety of ecological niches for microbes. The Healthy Food Diversity index (HFD-index) scores between 0 and 1–1/number of individuals (0.9998 for this study), where a higher value indicates a more diverse diet. The HFD-index was selected for this study as it considers diversity of food in conjunction with using a weighted health value to circumnavigate many of the traditional problems of measures of dietary diversity [22], and has been used in a previous microbiota study as a dietary covariate [23].

In this analysis, we first validate each index as a measure of a healthy diet within the TwinsUK cohort, and then asses each index’s association with measures of gut microbiota composition. Our aim is to determine the optimal summary measure of diet-based variation in gut microbiota composition for use as a covariate in future analyses.

## Results

All dietary indices were validated within the TwinsUK cohort, with results suggesting all three capture diet successfully. Microbiota associations were observed with all three indices, with the greatest number being associated with the HEI.

### Index construction and validation

Index scores created from data of 5047 individuals were used to assess index validity (Table 1). None of the indices achieved minimum or maximum scores possible in their 1st and 99th percentile, as expected given the real-world nature of the data (Additional file 1: Table S1). The range of all of the indices was wide enough to allow meaningful differences to be detected.

**Table 1:** Descriptive statistics of validation cohort and microbiota subset

Characteristic (measure)	Validation cohort	Microbiota subset
*n*	5047	2070
Sex (%female)	91.2	90
Zygosity (% MZ)	56.8	55.9
Ethnicity (% white)*	98.2	98.6
Age (at FFQ) (μ,σ^2^)	58.4 (13.2)	60.5 (11.5)
BMI (μ,σ^2^)*	26.2 (5)	25.9 (4.7)
FI (μ,σ^2^)*	0.2 (0.1)	0.19 (0.1)
HEI (μ,σ^2^)	60 (10.3)	60.4 (10.2)
MDS (μ,σ^2^)	4.6 (1.8)	4.5 (1.8)
HFD-index (μ,σ^2^)	0.2 (0.1)	0.2 (0.1)

Descriptive statistics of cohorts used to validate (validation cohort) three dietary indices and assess association with the microbiota (microbiota subset). Presented also are the means (μ) and standard deviation (σ^2^) of the three indices: the Healthy Eating Index (HEI), the Mediterranean Diet Score (MDS) and Healthy Food Diversity index (HFD-index). Zygosity is presented as % mono-zygotic twins (MZ), age as the date at which the Food Frequency Questionnaire (FFQ) was administered, body mass index by BMI (kg/m^2^), and frailty index (proportion of age-related health-deficits) as FI. *Data available on 88–90% subjects for these variables

Based on previous research, dietary indices are expected to be predictive of differences between populations known to have differing dietary patterns. In this case, concurrent criterion validation suggests that a dietary index predicts non-smokers, women and older people to have healthier diets than smokers, men and younger people, respectively [18]. All three indices significantly predicted a difference of means for smoking and non-smoking; the HEI and MDS for men and women, and just the MDS for age (Table 2).

**Table 2:** Concurrent criterion validation of dietary indices

	*n*	HEI	MDS	HFD-index
Men vs women	443:4604	56.4:60.4***	4.2:4.6***	0.2:0.19⊥
Over 60s vs under 60s	2543:2504	59.8:60.3⊥	4.5:4.6**	0.2:0.2⊥
Smokers vs non-smokers	317:2909	55.9:61***	4.1:4.7***	0.16:0.21***

Three dietary indices, the Healthy Eating Index (HEI), the Mediterranean Diet Score (MDS) and the Healthy Food Diversity index (HFD-index) were assessed for their ability to predict difference of diet of smokers vs non-smokers, over 60s vs under 60s, and men vs women via two sample t test (HFD-index via Wilcoxon rank sum). Difference in means is displayed for each grouping, with significance thresholds indicated by: **p < 0.01, ***p < 0.001, ⊥ = non-significant. Results of tests are indicated in Additional file 1: Table S2

Both the HEI and the MDS had a small, significant negative association with BMI; there was no significant association with the HFD-index (Table 3). HEI and MDS had a small but significant negative association with health as captured by the frailty index (where age, zygosity and sex were covariates). The frailty index (FI) is the proportion of age-related health deficits reported by subjects from over 30 holistic health domains [24]; the HFD-index exhibited a small positive association with FI suggesting that diversity of food is associated with adverse health (Table 3).

**Table 3:** Correlation of dietary indices with health measures

	*n*	HEI	MDS	HFD-index
BMI	4428	β = −0.076 ***	β = − 0.098***	Non-significant
FI	4553	β = −0.12***	β = − 0.11***	β = 0.013*

Three dietary indices, the Healthy Eating Index (HEI), the adjusted-Mediterranean Diet Score (MDS) and the Healthy Food Diversity index (HFD-index) were assessed for their correlation with two health measures; body mass index (BMI kg/m^2^) and Rockwood’s frailty index (FI) [24] via nested linear regression models (adjusting for age, sex and zygosity). *p* values: *p* < 0.05*, *p* < 0.001***

### Microbiota assessment

**Fig. 1:**
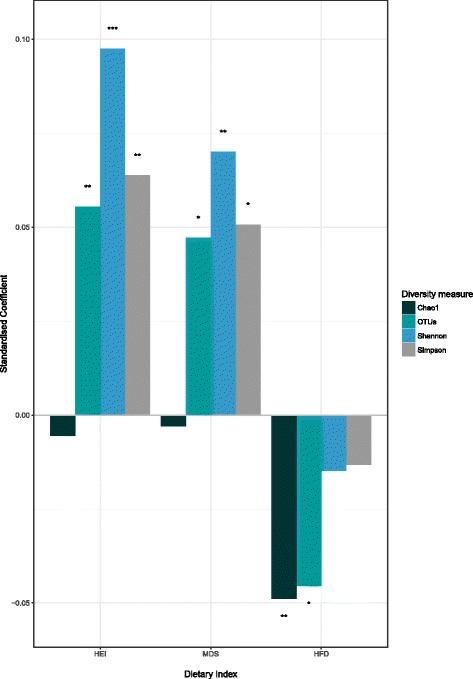
Standardised coefficients indicating correlation magnitude from mixed-effects models of three dietary indices (the Healthy Eating Index (HEI), Mediterranean Diet Score (MDS) and the Healthy Food Diversity index (HFD-index) for four measures of microbial alpha diversity; Chao1, observed OTUs, Shannon index and the Simpson index. Only significant results included, *p* values are *< 0.05, **< 0.01, ***< 0.001. Full results, including model AIC and *t* values are in Additional file 1: Table S3. Alpha diversity metrics were rarefied and adjusted for age, sex, gender and technical covariates

A subset of 2070 individuals with 16S rRNA gene sequencing gut microbiota data were used to assess the extent the dietary indices were able to explain variance within the cohorts microbial community structure (Table 1). Linear mixed-effects models were used to assess associations between the dietary indices and alpha diversity. All significant associations were small, with the highest β value observed between Shannon diversity and the HEI (Fig. 1, Table 4). The highest *t* values came from the HEI, where many were greater than 2, the threshold for indication of good model fit [25] (Additional file 1: Table S3). Both the HEI and MDS were significantly associated with number of OTUs, Shannon and Simpson indices; only the HFD-index was associated with diversity indicator Chao1. Interestingly, all alpha diversity associations with the HFD-index were negative. Comparison of *t* values from the HEI, MDS and HFD-index found that the HEI explains more of the variance within the data than the other two indices (Additional file 1: Table S3).

**Table 4:** Alpha diversity results

Diversity measure	HEI	MDS	HFD-Index
Chao1	−0.01⊥	> 0.00⊥	− 0.05**
OTUs	0.06**	0.05*	− 0.05*
Shannon	0.1***	0.07***	− 0.01⊥
Simpson	0.06**	0.05*	− 0.01⊥

Standardised coefficients of linear mixed-effects models of three dietary indices, the Healthy Eating Index (HEI), Mediterranean Diet Score (MDS) and the Healthy Food Diversity index (HFD-index), against four measures of alpha diversity (Chao1, Observed number of OTUs (OTUS), Shannon diversity and Simpson’s diversity index). Alpha diversity measures were rarefied and adjusted for BMI, sex, age and technical covariates (see the “Methods” section). *p* values: *p < 0.05, **p < 0.01, ***p < 0.001, ⊥ = non-significant

We used hierarchical modelling to investigate the contributions to variance explained by health and diet separately and together (Additional file 1: Table S4–6). Beta coefficients are similar across all models suggesting dietary indices capture alpha diversity variance attributable to diet independent of health deficits.

All three indices exhibited FDR-adjusted associations with individual OTU relative abundances significant at *q* < 0.05: the HEI had 167, the MDS had 107, and the HFD-index had 13 (Table 5, Additional file 1: Table S7–13). Both the HEI and MDS exhibited significant negative correlations with *Ruminococcus*, *Lachnospira* and *Actinomyce*s (Additional file 1: Table S9–13). The HFD-index also exhibited significant correlations with several *Ruminococcus* and *Lachnospiracae*; with only one genus-level association assigned to genus Cc115 within the family *Erysipelotrichaceae*.

**Table 5:** Number of taxonomic associations observed with dietary indices

Taxonomic level	HEI	MDS	HFD-index
OTUs	167	107	13
Genus	16	6	1
Phylum	4	0	0

Number of Qiime de novo derived operational taxonomic units (OTUs), genus and phyla significantly associated with three dietary indices, the Healthy Eating Index (HEI), Mediterranean Diet Score (MDS) and the Healthy Food Diversity index (HFD-index). Number of results are those significant post FDR adjustments in linear mixed effects models adjusted for age, BMI, sex and technical microbiota covariates. Full results are included in Additional file 1: Table S7–13

In linear mixed-effects models, the HEI was significantly associated with axes 1, 2, 4, 8 and 10 from PCoA of unweighted UniFrac distances; the MDS with the first 2 and the highest correlations for both was with axis 2 (HEI: β = − 0.14, *p* < 0.0001, MDS: β = − 0.12, *p* < 0.0001) (Additional file 1: Table S14–15). The HFD-index was approaching significance with axis 2 (β = − 0.039, *p* = 0.055) and axis 8 (β = − 0.095, *p* < 0.0001) (Additional file 1: Table S16).

**Fig. 2:**
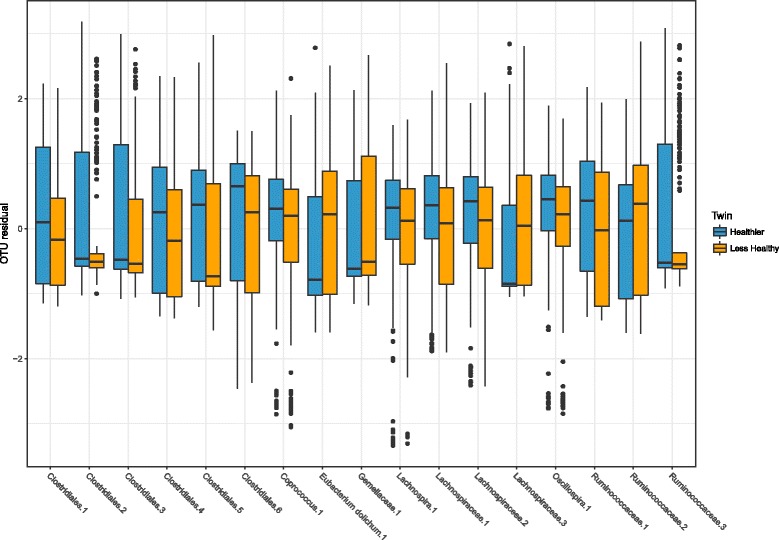
Box plot of OTU residuals (see the “Methods” section) significantly different between twins discordant for the Healthy Eating Index (HEI). Twins were characterised as healthy or less healthy relative to their co-twin if they were in differing HEI quantiles and their score differed by greater than 1 standard deviation (number of discordant twins pairs = 250). Of the 167 FDR-significant associations observed in mixed-effects models with the HEI, the 17 Qiime de novo derived operational taxonomic units (OTUs) presented here differed (FDR *q* < 0.05) between twin pairs in paired Wilcoxon rank-sum tests. *X* axis labels indicate the lowest taxonomic level assigned to each de novo OTU used in the analysis

The unique setting of this study within a large twin cohort allowed us to undertake twin paired tests that reduce the variation due to genetic and early-environmental factors. Twins discordant for their dietary index value were assessed using paired Wilcoxon rank-sum tests to replicate OTU associations. We observed that of the 167 HEI-associated OTUs, 71 were nominally significant in difference between “healthy diet” to “less healthy diet” pairs, and 17 were FDR-adjusted significant to *q* < 0.05 (Fig. 2). Of the 107 OTUs associated with the MDS, 32 were nominally significant and one, an OTU assigned to genus *Coprococcus*, was FDR significant. Of the 13 FDR-adjusted significant associations with the HFD-index, none were significantly associated with discordant twins. In regression analyses of weighted UniFrac distance between 755 twin pairs against dietary index dissimilarity, adjusted for difference of BMI and technical covariates, no significant associations were observed.

## Discussion

The primary aim in this analysis was to identify a dietary composite which explains variation in the gut microbiota, and therefore might have most utility to capture diet in microbiota studies. In this analysis, three dietary indices were successfully applied to FFQ data derived from the TwinsUK cohort and were assessed for their ability to explain inter-individual variance within the gut microbiota. Our evidence here is suggestive of the HEI being the index of choice.

We made some assumptions in this analysis; that it is the range of healthy diets along these indices that captures the greatest range of difference between microbial communities; that the dietary index that captures the highest amount of variance with measures of alpha and beta diversity, and the highest number of associations with OTUs is the index of preference. However, we make no assumption that the microbes associated with the higher dietary scores are necessarily the microbes that are the most important for health.

All three dietary indices performed in validation tests and could therefore have specific utility. All could distinguish between smokers and non-smokers; the HEI and MDS differed marginally between women and men; only the MDS could distinguish between young and old, but the magnitude of the effect was minimal. The HEI and MDS were significantly negatively associated with frailty as would be expected; the frailer a person, the less healthy their diet [26], further confirming validity of the HEI and MDS as a measure of healthy eating. Associations with health measures (BMI and frailty) were small, as expected due to the large number of factors influencing health [18, 24]. The positive HFD-index association with frailty, although small, was in an unexpected direction and is difficult to interpret. This may reflect the fact that the HFD-index was not calorie adjusted, whereas the other indices take total energy content into account. One concurrent criterion proposed by Guenther was that the Healthy Eating Index was improved in older adults compared with younger adults [18]. Our sample detected a small difference between age groups for the MDS, but no difference for the HEI. As the HEI is population independent, this may be a consequence of our study population demographics (older, white, middle-class women) with limited sampling in the younger age groups. The MDS could have succeeded here because it reflects dietary preference of specific food groups relative to the study population mean.

Our focus was to find a means of controlling for as much dietary-influenced microbiota variation as possible. Therefore, as the HEI had the greatest association with the microbiota and explained the most variance and dissimilarity of the data within this cohort, we argue that it can be deemed the most suitable index to use as a dietary covariate. Del Chierico 2014 [20] makes the a priori assumption that there will be compelling evidence for microbiota associations with the MDS based on its positive associations with health outcomes. Indeed, we observed, like others [19], the MDS to have associations with health measures and microbiota. However, the nature of the HEI (comprised of multifaceted components rather than binary variables and with a larger numeric range) means it covers more variation of diet compared with the MDS (with associations based on population medians and comprised of a much smaller numeric range). This may explain its larger capture of microbiota associations within our population.

The HFD-index also exhibited some intriguing associations as an index based on dietary variety; and the different outcomes when compared with the HEI and MDS may suggest some underlying associations driven by diversity of diet. However, its negative associations with alpha diversity are at odds with what might be hypothesised; that a more varied diet creates more ecological niches for a more diverse community assemblage. It is likely that patterns observed here are due to unsuitability of FFQ raw data for this index; many of the values used to create the health value were difficult to ascertain in quantities from the data (e.g. wheat germ oil and soy bean oil). Indeed, the FFQ has been designed to capture intakes of the most frequently consumed food for a population; therefore, inherent in the data is a limit on its ability to capture diversity of diet. Additionally, whilst diet-diversity indices are frequently utilised as indicators of nutrient intake in children and populations from lower-income countries [27, 28] they may be less suited to western diets.

The HEI can be appropriately applied to a wide range of dietary data types; particularly 24 h and 3-day recall diaries, and therefore offers opportunity as the covariate of choice for a wide range of microbiota studies [15, 18]. OTU associations that differed between twin pairs discordant for the HEI generally followed health-associated patterns previously observed. *Eubacterium dolichum,* associated with a lower (less-healthy) HEI score in the present study, was observed to positively associate with frailty [29] and with a dietary score based on visceral fat mass within this cohort [30]. This finding is in keeping with Murine models showing blooming of related bacteria (*Erysipelotrichi*) in the context of an unhealthy diet [5]; similarly, genus *Oscillospira* (here, associated positively with HEI) has been observed to be reduced in the presence of diseases that involve inflammation and patients with non-alcoholic fatty liver disease [31], and was negatively correlated with BMI differences in a different twin cohort [32]. *Clostridiales* are a polyphyletic group with some notable pathogenic gut species (e.g. *Clostridium difficile)*, yet contribute in force to the core microbiota [33]. Their decreased relative abundance has been shown to associate with disease states and here was enriched in twins with a higher HEI score relative to their sibling. Similarly, an increase in *Fusobacteria*was associated with disease states and was observed here in higher relative abundance in less healthy eaters [34]. *Lachnospiraceae* were less enriched in colorectal cancer patients and again here mostly associated with a higher HEI score [35]. Therefore, this suggests that the HEI is associated with bacterial species in a way that would be expected given its design as a measure of healthy diet, and is applicable as a means of explaining dietary impact on the community composition of the microbiota.

A key consideration in the utilisation of FFQ data is its appropriateness for the study population. The UK branch of the EPIC population, for which the FFQ used in the present study was derived, was deemed to be appropriate for this study because of similarities in population demographic. However, future studies should consider their study populations and adjust FFQs accordingly to capture regional and ethnic foods, as has been validated in [36–38], or add adjustment based on race and geography [39]. Furthermore, a key socioeconomic factor to consider in the interpretation of FFQ data is education status, as this has been shown to increase inaccuracy of the FFQ reporting [36, 40]. An adjusted FFQ that considers these factors could be used to create the HEI using methodology as described here, providing the use of an adequate food composition database. Alternatively, iterations of the HEI have been created and validated that better capture dietary data from other populations [16]. Similarly, the cohorts older age and majority female gender may impact how accurately FFQ captures the cohorts diet and therefore may influence the extent of variance captured by the HEI. Future studies should seek to confirm that with adjustments to the HEI, it remains the most appropriate index of choice across different populations.

A drawback of using any self-reported diet data is that individuals have a tendency to inaccurately report their consumption of food items; generally, over reporting fruits and vegetables and under reporting food items that are considered unhealthy [41, 42]. Drawing direct associations between a disease and individual dietary components derived from FFQs has been shown to be problematic [41], and points to the strength of comparative summary measures. Another consideration is that FFQ is designed to measure long-term habitual food intakes, whereas the eating behaviour immediately before microbiota sampling may diverge from typical eating behaviour. Although a lasting shift in community structure is unlikely from short-term changes [43], future studies should also explore and consider secondary adjustments to capture short-term food intakes.

The HEI as presented here had some limitations. The HEI (and MDS) were both developed in countries outside of this cohort, and an index created using UK-specific thresholds of consumption may have performed even better within this population. The HEI might also benefit from the use of a diversity measure as one of the components as has been done in adjusted HEI studies [17, 44].

The benefits of a healthy diet are well known [45], and it is important to note that a healthy diet-associated microbiota may not directly drive these outcomes or be directly influenced as a result of dietary consumption. These are also influenced by indirect effects associated with a healthy lifestyle [46]. Many of the microbial associations observed here were small. This is possibly reflective of the complex intertwining factors affecting the community composition of the microbiota. However, until the nuances of these relationships with the microbiota are fully characterised, the HEI offers an effective way of capturing wider dietary information in a single, weighted, energy-adjusted variable when other factors are of interest.

The HEI is likely inappropriate as a predictor variable of differences between microbial ecosystems as poor diets that are different but score similarly may mask trends due to specific dietary constituents. Future studies should expand on existing work to probe the effect of specific dietary elements on microbiota, but in these studies, it will be important to co-vary for overall dietary health. This study supports the use of an HEI approach in such endeavours.

## Conclusions

Of the indices studied here, associations with measures of gut microbiota composition show the Heathy Eating Index (HEI) as the most appropriate, previously established, dietary index to utilise as a covariate in microbiota studies within this population. Adjustment of thresholds or FFQ parameters could readily be applied in different demographics, but would need to be tested. As a single variable, it is readily applied to a wide range of dietary data, has extensive resources provided by the USDA to aid its analysis and creation and is readily adjustable and interpretable. This will allow future research to adequately control for diet without saturation of models by the high dimensionality of diet data, allowing researchers to better interpret the effect of other environmental factors on the gut microbiota and potentially other human-microbe interaction models which necessitate adjustment for diet.

## Methods

### Study population

All individuals included in this study are part of the TwinsUK research cohort, the UK’s largest research database of mono- and dizygotic twins. Descriptive statistics for the data used here are provided in Table 1.

### Food frequency questionnaire (FFQ) data

Food frequency questionnaire (FFQ) data was collected following the EPIC-Norfolk guidelines [47], with only those answering for all 152 food groups considered for this analysis. As with any ongoing large-cohort study, the data was collected in batches; both undertaken on rolling bases The first was undertaken predominantly in 2007 for 3370 individuals, and the second between 2014 and 2015 for 4116; 5047 unique individuals were used here. All analysis considered the score for the nearest time-point, excluding subjects with a greater than 5-year difference; subsequent microbiota analysis was undertaken on data matched to samples from 2070 individuals.

### HEI construction

All indices were constructed in RStudio [48] following relevant methodologies. The HEI was constructed following Guenther et al. 2013 guidelines [15]. The reported weekly frequency of each FFQ food item was converted to the unit recommended by the HEI guidelines (Additional file 1: Table S17). Divergences from the published methodology was the use of the ‘*Composition of foods integrated database’* (CoFids) published by Public Health England [49], as a more appropriate look-up table for a UK-based cohort. CoFids was used where calories and proportional components of FFQ food items were required for calculating HEI components (Additional file 1: Table S17). Where available, volumetric conversions were calculated using specific gravities obtained from the USDA websites. Similar to subsequent studies [17, 50], food items were categorised into HEI components based on their predominant attributes (e.g. broken down into lean and solid fat fractions) after being initially classified into the USDA sub-groupings, rather than being considered within all groups (Additional file 1: Table S17.)

### MDS construction

There is some variance in methodology in assigning the MDS depending on the different weighting of evidence for factors considered to constitute a healthy diet [19, 51]; here, the MDS was constructed using the modified MDS methodology outlined by Trichopoulou et al. 2005 [19]. Estimates of daily grammes of consumption were created from residual energy-adjusted FFQ data of seven groups (Additional file 1: Table S18). Scores were assigned to each category as either 0 (no MDS) or 1 (MDS) for each category depending on whether the twin was above or below median intake of the study population. Medians were calculated using the combined scores for each FFQ ‘batch’.

### HFD-index construction

Methodology from Drescher et al. 2007 [22] was used to create this index, where a healthy food value is calculated for each FFQ food item and used as a weighting for multiplication against a Simpson’s index score of all consumed foods, indicating the diversity (Additional file 1: Table S19). This results in a diversity measure of diet that considers the health value of the variety of foods combined.

### Validation of indices

All statistical analysis was undertaken in Rstudio. Construct validity of indices was assessed following partial methodology from Guenther et al. 2014 [18]. First, via review of overall distributions of the total index score. Secondly, as healthy diets distinguish smokers from non-smokers, young from old and men from women, concurrent criterion validity was assessed using two sample *t* tests for the MDS and HEI, where distributions approached normalcy, and Wilcoxon. Age was calculated as age at questionnaire submission and separated into two groups; below 50s and over 50s, and those who self-reported as current smokers used to assess differences between smokers and non-smokers. 5047 individuals were used to assess age and sex differences; due to data absent due to longitudinal differences in data collection, a subset of 3226 were used to assess differences between smokers and non-smokers.

Indices were also assessed as the primary explanatory variable against health measures in nested linear regression models with age, twin zygosity, and sex as covariates against BMI on a subset of 4428 individuals missing data, again due to differences in collection method. Similarly, on a subset of 4553 individuals following the Rockwood method [24], a frailty index of the TwinsUK participants was used to indicate the health predictive capacity of each dietary indices, zygosity and sex as covariates.

### Microbiota analysis

A subset of 2070 individuals were used to assess the extent the variation within and between individuals’ microbiota could be captured by each dietary index. Collection and processing of samples for 16S rRNA gene sequencing for the TwinsUK cohort has been described previously [52]. Individuals brought samples to clinical visit or posted them in sealed ice packs to the research department where they were stored at − 80 °C, until shipped frozen for analysis. DNA was extracted at Cornell University, where the V4 region of the 16S rRNA genes was amplified. A multiplexed approach was used to sequence the amplicons on the Illumina MiSeq platform. Following demultiplexing, sample read paired-ends were merged using a 200 nt minimum overlap. 16S rRNA gene sequencing data was processed and OTUs generated as described previously [53]; per sample de novo identification and removal of chimeric sequences was undertaken using USEARCH, and then de novo OTUs were picked in QIIME using SUMACLUST at a similarity threshold of 97% [54]. The OTU representative sequences were aligned using the parallel_align_seqs_pynast command within QIIME, the resulting alignment was then filtered to remove variable regions using the filter_alignment command, and a phylogenetic tree was created using the make_phylogeny command. All commands were run with the default parameters in QIIME version 1.9.1.

Alpha diversity metrics of Shannon diversity, chao1, Simpson’s diversity and observed species were also calculated in Qiime. OTUs were rarefied to 10,000 sequences per sample 50 times, and the 4 alpha diversity metrics were then calculated as the mean for each sample across the 50 rarefied tables. Mixed-effects models were constructed using the “lme4” package in R to assess the extent alpha diversity varied with dietary index; all model variables were scaled prior to input, and all reported coefficients are standardised [25]. Nested models were used to compare the effect of each dietary index. Models were adjusted for age, BMI, twin zygosity, sex and OTU count per samples, with technical covariates and FFQ questionnaire batch as random effects. As *χ*^2^ values resulting from ANOVA of two mixed models are only appropriate for comparisons of nested models, to assess relative goodness of fit of the three dietary indices, *t* values, AICs and β coefficients from the mixed-effects models for each index were used to quantify the ability of a dietary index to capture each measure. To further assess the ability of dietary indices to capture variance, hierarchical models of alpha diversity were performed with BMI and a smaller subset (*n* = 2015) incorporating frailty data.

Relative abundances of OTUs found in > 25% in individuals were log10 transformed, and residuals were generated via regression against technical covariates of sequencing depth, sequence run, person who extracted the DNA, person who loaded the DNA and sample collection method. OTUs were collapsed to taxonomic abundances and Family and Genus levels. All OTU metrics were used as response variables in mixed-effects models (as above) adjusted for age, twin zygosity, BMI and sex, with FFQ batch as a random effect. Nested models were compared using ANOVA, and *p* values were false discovery rate (FDR) adjusted using the *q*value package [55]. Twin pairs discordant by greater than one standard deviation and within different quartiles were identified, and OTU differences between the two were assessed using paired Wilcoxon rank-sum tests and FDR adjustment.

Unweighted UniFrac distances were calculated as β diversity measures using the phyloseq package in R [56]. Ordination plots were also generated using phyloseq, and the first 10 components from the PCoA (representing the first 10 axes) were extracted and used as the response variable in mixed-effects models, as in alpha diversity analysis. Finally, weighted UniFrac distances between twin pairs were used as the response variables in regression models with difference in dietary index, difference in BMI, and differences in factorial technical variables (person who extracted the DNA, person who loaded the DNA and sample collection method) as covariates. Standardised coefficients were calculated using the lm.beta package [57].

## Additional files

Additional file 1: Supplementary tables. (DOCX 167 kb)

Additional file 2: HEI creation. (ZIP 233 kb)
