# Supplementary material for: Use of dietary indices to control for diet in human gut microbiota studies
Source: Microbiome. 2018 Apr 25;6:77. doi: 10.1186/s40168-018-0455-y (PMC5918560; doi:10.1186/s40168-018-0455-y)
Supplement: Supplementary file 1 — Supplementary tables. (DOCX 167 kb) [file 40168_2018_455_MOESM1_ESM.docx]

**Additional file 1: Table S1**. Percentiles and means of the three dietary indices ; the Healthy Eating Index (HEI), Mediterranean Diet Score (MDS) and the Healthy Food Diversity index (HFD-index).

|  |  | Percentile | | | | | | | | |
| --- | --- | --- | --- | --- | --- | --- | --- | --- | --- | --- |
|  | Mean +/- SE | 1^st^ | 5^th^ | 10^th^ | 25^th^ | 50^th^ | 75^th^ | 90^th^ | 95^th^ | 99^th^ |
| HEI | 60.03 +/- 0.16 | 37 | 43 | 47 | 53 | 60 | 67 | 73 | 77 | 86 |
| MDS | 4.55 +/- 0.03 | 1 | 2 | 2 | 3 | 5 | 6 | 7 | 7 | 8 |
| HFD-index | 0.207 +/- 0.002 | 0.05 | 0.08 | 0.1 | 0.14 | 0.19 | 0.25 | 0.35 | 0.41 | 0.56 |

Additional file 1: Table S2: Three dietary indices, the healthy eating index (HEI), the Mediterranean Diet Score (MDS) and the healthy food diversity index (HFD-index) were assessed for their ability to predict difference of diet of smokers vs non-smokers, over 60s vs under 60s, and men vs women via two sample t-test (HFD-index via wilcoxon rank sum). Results of tests are indicated below, with significance thresholds indicated by *p < 0.05, **p < 0.01, ***p <0.001, ⊥ = Non significant

|  | n | HEI | MDS | HFD-index |
| --- | --- | --- | --- | --- |
| Men vs women | 443:4604 | t(532.21)= 7.9*** | t(540.43)= 5.0873*** | W = 1067300 ⊥ |
| Over 60s vs Under 60s | 2543:2504 | t(5032) = -1.5 | t(5042.7)=-3.2** | W = 3208900 ⊥ |
| Smokers vs non-smokers | 317:2909 | t(376)= -7.9013*** | t(386.45)= -5.4753*** | W = 590270*** |

**Additional file 1: Table S3 .** Results of mixed effects models of three dietary indices (the Healthy Eating Index (HEI), Mediterranean Diet Score (MDS) and the Healthy Food Diversity index (HFD-index) against four measures of alpha diversity (Choa1, Observed number of OTUs, Shannon diversity and Simpson's diversity index). Alpha diversity measures were rarefied and adjusted for BMI, sex, age and technical covariates (see methods).

| **Measure of Alpha Diversity** | **AIC value** | **T-value** | **Standardised coefficient** | **P value** |
| --- | --- | --- | --- | --- |
| *Healthy Eating Index* | | | |  |
| Chao 1 | 4507.346709 | -0.359101876 | -0.005569664 | 0.719590509 |
| Observed OTUs | 5263.666363 | 2.953861501 | 0.055378125 | **0.003181448** |
| Shannon | 5804.526325 | 4.502957646 | 0.097482145 | **6.00E-06** |
| Simpson | 5862.262319 | 2.902652575 | 0.0637337 | **0.003754278** |
| *Mediterranean Dietary Index* | | | |  |
| Chao 1 | 4505.437394 | -0.195482144 | -0.00303886 | 0.845042625 |
| Observed OTUs | 5264.119264 | 2.50724045 | 0.04714808 | **0.012403445** |
| Shannon | 5812.359736 | 3.216645186 | 0.070002336 | **0.001097205** |
| Simpson | 5863.385771 | 2.299012038 | 0.050688632 | **0.021626997** |
| *Healthy Food Diversity* | | | | |
| Chao 1 | 4495.418334 | -3.175682575 | -0.04893136 | **0.001517488** |
| Observed OTUs | 5264.447696 | -2.434665241 | -0.045474723 | **0.014978107** |
| Shannon | 5822.229444 | -0.686699203 | -0.014870864 | 0.50241552 |
| Simpson | 5868.300091 | -0.602404032 | -0.013187754 | 0.547826139 |

**Additional file 1: Table S4a.** Model AIC values and Beta coefficients of fixed effects in mixed effects models of four measures of alpha diversity (Chao1, Observed OTUs, Shannon diversity and Simpsons diversity), adjusted for (in microbiota subset): i. BMI, age and technical covariates, ii. Measure of dietary quality the Healthy Eating Index (HEI) and technical covariates and iii. HEI, BMI, age and technical covariates;

**4b** In subset of 2015 individuals with frailty data; iv frailty, age, and technical covariates, v. HEI, frailty, age and technical covariates and vi HEI, BMI, frailty, age and technical covariates.

Age is considered as age at microbiota sample, Body Mass Index is indicated as BMI (kg/m^2^) and frailty index in noted by FI.

| **A.** | **Microbiota subset ( n =2070)** | | | | | | | | | | | |
| --- | --- | --- | --- | --- | --- | --- | --- | --- | --- | --- | --- | --- |
|  | **Model i - Age + BMI** | | | | **Model ii - HEI** | | | | **Model iii - HEI + Age + BMI** | | | |
|  | **Chao1** | **OTUs** | **Shannon** | **Simpson** | **Chao1** | **OTUs** | **Shannon** | **Simpson** | **Chao1** | **OTUs** | **Shannon** | **Simpson** |
| *AIC value:* | *4503.48* | *5268.37* | *5820.68* | *5866.66* | *4512.13* | *5328.51* | *5855.53* | *5874.83* | *4507.35* | *5263.67* | *5804.53* | *5862.26* |
| Age | 0.018 | **0.08***** | **0.089***** | **0.051*** | - | - | - | - | 0.017 | **0.08***** | **0.09***** | **0.051*** |
| BMI | **-0.044**** | **-0.149***** | **-0.154***** | **-0.085***** | - | - | - | - | **-0.045**** | **-0.143***** | **-0.143***** | **-0.078***** |
| HEI | - | - | - | - | -0.001 | **0.069***** | **0.111***** | **0.071**** | -0.006 | **0.055***** | **0.097***** | **0.06**** |
|  | | | | | | | | | | | | |
| **B.** | **Frailty subset (n = 2015)** | | | | | | | | | | | |
|  | **Model iv - Age + BMI + FI** | | | | **Model v - HEI + FI + Age** | | | | **Model vi - HEI + Age + BMI + FI** | | | |
|  | **Chao1** | **OTUs** | **Shannon** | **Simpson** | **Chao1** | **OTUs** | **Shannon** | **Simpson** | **Chao1** | **OTUs** | **Shannon** | **Simpson** |
| *AIC value:* | *4452.74* | *5129.76* | *5621.75* | *5690.27* | *4456.82* | *5154.04* | *5628.40* | *5690.86* | *4454.64* | *5125.69* | *5611.91* | *5688.10* |
| Age | 0.029 | **0.119***** | **0.136***** | **0.083***** | 0.029 | **0.119***** | **0.136***** | **0.082***** | 0.029 | **0.119***** | **0.136***** | **0.083***** |
| BMI | **-0.034*** | **-0.115***** | **-0.105***** | **-0.054*** | - | - | - | - | **-0.034*** | **-0.111***** | **-0.099***** | **-0.051*** |
| FI | **-0.048**** | **-0.139***** | **0.179***** | **-0.126***** | **-0.06***** | **-0.17***** | **-0.205***** | **-0.136***** | **-0.049**** | **-0.134***** | **-0.172***** | **-0.122***** |
| HEI | - | - | - | - | -0.003 | **0.056**** | **0.083***** | **0.049*** | -0.005 | **0.047*** | **0.075***** | **0.045*** |

**Additional file 1: Table S5a** Model AIC values and Beta coefficients of fixed effects in mixed effects models of four measures of alpha diversity (Chao1, Observed OTUs, Shannon diversity and Simpsons diversity), adjusted for (in microbiota subset): i. BMI, age and technical covariates, ii. Measure of dietary quality the Mediterranean Dietary Score (MDS) and technical covariates and iii. MDS, BMI, age and technical covariates;

**5b.** In subset of 2015 individuals with frailty data; iv frailty, age, and technical covariates, v. MDS, frailty, age and technical covariates and vi. MDS, BMI, frailty, age and technical covariates.

Age is considered as age at microbiota sample, Body Mass Index is indicated as BMI (kg/m^2^) and frailty index in noted by FI.

| **A.** | **Microbiota subset ( n =2070)** | | | | | | | | | | | |
| --- | --- | --- | --- | --- | --- | --- | --- | --- | --- | --- | --- | --- |
|  | **Model i - Age + BMI** | | | | **Model ii - MDS** | | | | **Model iii - MDS + Age + BMI** | | | |
|  | **Chao1** | **OTUs** | **Shannon** | **Simpson** | **Chao1** | **OTUs** | **Shannon** | **Simpson** | **Chao1** | **OTUs** | **Shannon** | **Simpson** |
| *AIC value:* | *4503.48* | *5268.37* | *5820.68* | *5866.66* | *4512.09* | *5329.33* | *5864.96* | *5877.56* | *4507.44* | *5266.11* | *5814.36* | *5865.39* |
| Age | 0.018 | **0.08***** | **0.089***** | **0.051*** | - | - | - | - | 0.018 | **0.08***** | **0.089***** | **0.051*** |
| BMI | **-0.044**** | **-0.149***** | **-0.154***** | **-0.085***** | - | - | - | - | **-0.044**** | **-0.142***** | **-0.144***** | **-0.078***** |
| MDS | - | - | - | - | 0.003 | **0.067***** | **0.089***** | **0.061**** | -0.003 | **0.047*** | **0.070***** | **0.051*** |
|  | | | | | | | | | | | | |
| **B.** | **Frailty subset (n = 2015)** | | | | | | | | | | | |
|  | **Model iv - Age + BMI + FI** | | | | **Model v - MDS + FI + Age** | | | | **Model vi - MDS + Age + BMI + FI** | | | |
|  | **Chao1** | **OTUs** | **Shannon** | **Simpson** | **Chao1** | **OTUs** | **Shannon** | **Simpson** | **Chao1** | **OTUs** | **Shannon** | **Simpson** |
| *AIC value:* | *4452.74* | *5129.76* | *5621.75* | *5690.27* | *4456.57* | *5153.82* | *5632.93* | *5691.72* | *4454.66* | *5126.44* | *5616.87* | *5689.16* |
| Age | 0.029 | **0.119***** | **0.136***** | **0.083***** | 0.029 | **0.118***** | **0.136***** | **0.081***** | 0.029 | **0.118***** | **0.135***** | **0.082***** |
| BMI | **-0.034*** | **-0.115***** | **-0.105***** | **-0.054*** | - | - | - | - | **-0.033*** | **-0.109***** | **-0.098***** | **-0.05*** |
| FI | **-0.048**** | **-0.139***** | **0.179***** | **-0.126***** | **-0.058***** | **-0.169***** | **-0.206***** | **-0.136***** | **-0.048**** | **-0.135***** | **-0.174***** | **-0.122***** |
| MDS | - | - | - | - | 0.008 | **0.056**** | **0.069**** | **0.045*** | 0.005 | **0.044*** | **0.057**** | 0.039 |

**Additional file 1: Table S6a** Model AIC values and Beta coefficients of fixed effects in mixed effects models of four measures of alpha diversity (Chao1, Observed OTUs, Shannon diversity and Simpsons diversity), adjusted for (in microbiota subset): i. BMI, age and technical covariates, ii. Measure of dietary quality the Healthy Food Diversity Index (HFD-Index) and technical covariates and iii. HFD-index, BMI, age and technical covariates;

**6b.** In subset of 2015 individuals with frailty data; iv frailty, age, and technical covariates, v. HFD-index, frailty, age and technical covariates and vi. HFD-index, BMI, frailty, age and technical covariates.

Age is considered as age at microbiota sample, Body Mass Index is indicated as BMI (kg/m^2^) and frailty index in noted by FI.

| **A.** | **Microbiota subset ( n =2070)** | | | | | | | | | | | |
| --- | --- | --- | --- | --- | --- | --- | --- | --- | --- | --- | --- | --- |
|  | **Model i - Age + BMI** | | | | **Model ii - HFD** | | | | **Model iii - HFD + Age + BMI** | | | |
|  | **Chao1** | **OTUs** | **Shannon** | **Simpson** | **Chao1** | **OTUs** | **Shannon** | **Simpson** | **Chao1** | **OTUs** | **Shannon** | **Simpson** |
| *AIC value:* | *4503.48* | *5268.37* | *5820.68* | *5866.66* | *4502.94* | *5337.44* | *5881.42* | *5885.18* | *4497.42* | *5266.45* | *5824.55* | *5870.30* |
| Age | 0.018 | **0.08***** | **0.089***** | **0.051*** | - | - | - | - | 0.016 | 0.078 | **0.088***** | **0.051*** |
| BMI | **-0.044**** | **-0.149***** | **-0.154***** | **-0.085***** | - | - | - | - | **-0.047**** | **-0.152***** | **-0.155***** | **-0.086***** |
| HFD - index | - | - | - | - | **-0.047**** | **-0.039*** | -0.009 | -0.010 | **-0.049**** | **-0.045*** | -0.015 | -0.013 |
|  | | | | | | | | | | | | |
| **B.** | **Frailty subset (n = 2015)** | | | | | | | | | | | |
|  | **Model iv - Age + BMI + FI** | | | | **Model v - HFD + FI + Age** | | | | **Model vi - HFD + Age + BMI + FI** | | | |
|  | **Chao1** | **OTUs** | **Shannon** | **Simpson** | **Chao1** | **OTUs** | **Shannon** | **Simpson** | **Chao1** | **OTUs** | **Shannon** | **Simpson** |
| *AIC value:* | *4452.74* | *5129.76* | *5621.75* | *5690.27* | *4448.95* | *5159.63* | *5642.72* | *5695.58* | *4446.09* | *5127.55* | *5623.51* | *5691.89* |
| Age | 0.029 | **0.119***** | **0.136***** | **0.083***** | 0.027 | 0.118*** | **0.137***** | **0.082***** | 0.027 | **0.117***** | **0.136***** | 0.083*** |
| BMI | **-0.034*** | **-0.115***** | **-0.105***** | **-0.054*** | - | - | - | - | **-0.037*** | **-0.117***** | **-0.106***** | -0.055* |
| FI | **-0.048**** | **-0.139***** | **0.179***** | **-0.126***** | **-0.059***** | **-0.177***** | **-0.215***** | **-0.142***** | **-0.047**** | **-0.138***** | **-0.178***** | -0.126*** |
| HFD - index | - | - | - | - | **-0.045**** | -0.032 | -0.004 | -0.010 | **-0.047**** | **-0.039*** | -0.011 | -0.014 |

**Additional file 1: Table S7**. Results of mixed effects models of OTU associations with the Healthy Eating Index (see methods). Only FDR significant results listed, Number of significant results = 167

| **OTU** | **Beta** | **Nom_P** | **FDR.P** | **Assigned.Taxonomy** |
| --- | --- | --- | --- | --- |
| denovo102 | 0.160331831 | 1.89E-13 | 5.88E-07 | k__Bacteria; p__Firmicutes; c__Clostridia; o__Clostridiales; f__Ruminococcaceae; g__Oscillospira; s__ |
| denovo27 | -0.138749129 | 2.14E-10 | 5.88E-07 | k__Bacteria; p__Firmicutes; c__Clostridia; o__Clostridiales; f__Lachnospiraceae; g__[Ruminococcus]; s__gnavus |
| denovo277 | 0.176282549 | 5.51E-16 | 5.88E-07 | k__Bacteria; p__Firmicutes; c__Clostridia; o__Clostridiales; f__; g__; s__ |
| denovo284 | 0.127186869 | 5.86E-09 | 5.88E-07 | k__Bacteria; p__Firmicutes; c__Clostridia; o__Clostridiales; f__Ruminococcaceae; g__; s__ |
| denovo348 | 0.130231799 | 2.72E-09 | 5.88E-07 | k__Bacteria; p__Firmicutes; c__Clostridia; o__Clostridiales; f__Ruminococcaceae; g__; s__ |
| denovo365 | 0.131030418 | 2.19E-09 | 5.88E-07 | k__Bacteria; p__Firmicutes; c__Clostridia; o__Clostridiales; f__[Mogibacteriaceae]; g__; s__ |
| denovo375 | 0.134791125 | 7.07E-10 | 5.88E-07 | k__Bacteria; p__Firmicutes; c__Clostridia; o__Clostridiales; f__; g__; s__ |
| denovo526 | 0.128265599 | 3.36E-09 | 5.88E-07 | k__Bacteria; p__Firmicutes; c__Clostridia; o__Clostridiales; f__Ruminococcaceae; g__Oscillospira; s__ |
| denovo55 | -0.129206954 | 3.20E-09 | 5.88E-07 | k__Bacteria; p__Firmicutes; c__Clostridia; o__Clostridiales; f__Lachnospiraceae; g__[Ruminococcus]; s__ |
| denovo56 | 0.141015271 | 8.61E-11 | 5.88E-07 | k__Bacteria; p__Firmicutes; c__Clostridia; o__Clostridiales; f__Lachnospiraceae; g__; s__ |
| denovo57 | 0.125234668 | 6.69E-09 | 5.88E-07 | k__Bacteria; p__Firmicutes; c__Clostridia; o__Clostridiales; f__Lachnospiraceae; g__Lachnospira; s__ |
| denovo604 | 0.140129105 | 1.73E-10 | 5.88E-07 | k__Bacteria; p__Firmicutes; c__Clostridia; o__Clostridiales; f__; g__; s__ |
| denovo753 | -0.140996468 | 1.01E-10 | 5.88E-07 | k__Bacteria; p__Firmicutes; c__Clostridia; o__Clostridiales; f__Lachnospiraceae; g__; s__ |
| denovo79 | 0.125818304 | 1.05E-08 | 6.14E-07 | k__Bacteria; p__Firmicutes; c__Clostridia; o__Clostridiales; f__Ruminococcaceae; g__; s__ |
| denovo428 | 0.123416015 | 1.69E-08 | 9.07E-07 | k__Bacteria; p__Firmicutes; c__Clostridia; o__Clostridiales; f__Peptococcaceae; g__; s__ |
| denovo364 | -0.124012068 | 1.78E-08 | 9.48E-07 | k__Bacteria; p__Firmicutes; c__Clostridia; o__Clostridiales; f__Lachnospiraceae; g__[Ruminococcus]; s__ |
| denovo469 | 0.121511861 | 2.70E-08 | 1.34E-06 | k__Bacteria; p__Firmicutes; c__Clostridia; o__Clostridiales; f__Ruminococcaceae; g__Faecalibacterium; s__prausnitzii |
| denovo552 | 0.121147313 | 2.78E-08 | 1.38E-06 | k__Bacteria; p__Firmicutes; c__Clostridia; o__Clostridiales; f__Christensenellaceae; g__; s__ |
| denovo482 | 0.121228436 | 2.95E-08 | 1.45E-06 | k__Bacteria; p__Firmicutes; c__Clostridia; o__Clostridiales; f__; g__; s__ |
| denovo123 | 0.120526104 | 3.17E-08 | 1.53E-06 | k__Bacteria; p__Firmicutes; c__Clostridia; o__Clostridiales; f__Ruminococcaceae; g__; s__ |
| denovo383 | 0.119203061 | 5.03E-08 | 2.27E-06 | k__Bacteria; p__Firmicutes; c__Clostridia; o__Clostridiales; f__Lachnospiraceae; g__; s__ |
| denovo467 | -0.118263246 | 6.97E-08 | 3.00E-06 | k__Bacteria; p__Actinobacteria; c__Actinobacteria; o__Actinomycetales; f__Actinomycetaceae; g__Actinomyces; s__ |
| denovo313 | 0.118037525 | 7.46E-08 | 3.19E-06 | k__Bacteria; p__Firmicutes; c__Clostridia; o__Clostridiales; f__Lachnospiraceae; g__; s__ |
| denovo151 | 0.115938184 | 1.02E-07 | 4.15E-06 | k__Bacteria; p__Firmicutes; c__Clostridia; o__Clostridiales; f__; g__; s__ |
| denovo13 | -0.115634823 | 1.22E-07 | 4.86E-06 | k__Bacteria; p__Firmicutes; c__Clostridia; o__Clostridiales; f__Lachnospiraceae; g__Blautia; s__ |
| denovo1432 | 0.114149069 | 1.96E-07 | 7.36E-06 | k__Bacteria; p__Firmicutes; c__Clostridia; o__Clostridiales; f__; g__; s__ |
| denovo1220 | 0.111136678 | 3.67E-07 | 1.27E-05 | k__Bacteria; p__Firmicutes; c__Clostridia; o__Clostridiales; f__; g__; s__ |
| denovo344 | 0.111322683 | 3.83E-07 | 1.32E-05 | k__Bacteria; p__Firmicutes; c__Clostridia; o__Clostridiales; f__Ruminococcaceae; g__; s__ |
| denovo129 | 0.111295257 | 3.84E-07 | 1.32E-05 | k__Bacteria; p__Firmicutes; c__Clostridia; o__Clostridiales; f__Lachnospiraceae; g__Lachnospira; s__ |
| denovo278 | -0.110668402 | 4.90E-07 | 1.63E-05 | k__Bacteria; p__Firmicutes; c__Erysipelotrichi; o__Erysipelotrichales; f__Erysipelotrichaceae; g__[Eubacterium]; s__dolichum |
| denovo33 | 0.110631222 | 4.96E-07 | 1.65E-05 | k__Bacteria; p__Firmicutes; c__Clostridia; o__Clostridiales; f__Ruminococcaceae; g__; s__ |
| denovo299 | 0.107315344 | 7.87E-07 | 2.47E-05 | k__Bacteria; p__Firmicutes; c__Clostridia; o__Clostridiales; f__; g__; s__ |
| denovo72 | 0.106772418 | 8.94E-07 | 2.76E-05 | k__Bacteria; p__Firmicutes; c__Clostridia; o__Clostridiales; f__; g__; s__ |
| denovo197 | 0.105505574 | 1.29E-06 | 3.80E-05 | k__Bacteria; p__Firmicutes; c__Clostridia; o__Clostridiales; f__Clostridiaceae; g__; s__ |
| denovo59 | 0.10539235 | 1.32E-06 | 3.86E-05 | k__Bacteria; p__Firmicutes; c__Clostridia; o__Clostridiales; f__Ruminococcaceae; g__Oscillospira; s__ |
| denovo242 | 0.105868728 | 1.35E-06 | 3.96E-05 | k__Bacteria; p__Firmicutes; c__Clostridia; o__Clostridiales; f__Lachnospiraceae; g__Coprococcus; s__ |
| denovo90 | 0.105073427 | 1.37E-06 | 3.99E-05 | k__Bacteria; p__Firmicutes; c__Clostridia; o__Clostridiales; f__Lachnospiraceae; g__Coprococcus; s__ |
| denovo336 | 0.105360961 | 1.54E-06 | 4.44E-05 | k__Bacteria; p__Firmicutes; c__Clostridia; o__Clostridiales; f__Ruminococcaceae; g__Oscillospira; s__ |
| denovo512 | -0.105702707 | 1.60E-06 | 4.59E-05 | k__Bacteria; p__Firmicutes; c__Clostridia; o__Clostridiales; f__[Mogibacteriaceae]; g__; s__ |
| denovo276 | 0.103302842 | 1.85E-06 | 5.20E-05 | k__Bacteria; p__Firmicutes; c__Clostridia; o__Clostridiales; f__Ruminococcaceae; g__Oscillospira; s__ |
| denovo88 | -0.104837723 | 1.90E-06 | 5.32E-05 | k__Bacteria; p__Firmicutes; c__Clostridia; o__Clostridiales; f__Ruminococcaceae; g__; s__ |
| denovo110 | 0.102729612 | 1.99E-06 | 5.54E-05 | k__Bacteria; p__Firmicutes; c__Clostridia; o__Clostridiales; f__Ruminococcaceae; g__; s__ |
| denovo212 | 0.102966358 | 2.61E-06 | 7.01E-05 | k__Bacteria; p__Firmicutes; c__Clostridia; o__Clostridiales; f__Ruminococcaceae; g__; s__ |
| denovo109 | 0.10176745 | 3.02E-06 | 7.95E-05 | k__Bacteria; p__Firmicutes; c__Clostridia; o__Clostridiales; f__; g__; s__ |
| denovo958 | 0.101211018 | 3.20E-06 | 8.36E-05 | k__Bacteria; p__Firmicutes; c__Clostridia; o__Clostridiales; f__; g__; s__ |
| denovo1116 | 0.10053073 | 3.60E-06 | 9.26E-05 | k__Bacteria; p__Firmicutes; c__Clostridia; o__Clostridiales; f__; g__; s__ |
| denovo381 | 0.100151099 | 4.08E-06 | 0.000103044 | k__Bacteria; p__Firmicutes; c__Clostridia; o__Clostridiales; f__Ruminococcaceae; g__; s__ |
| denovo1898 | 0.101383366 | 4.16E-06 | 0.000104735 | k__Bacteria; p__Firmicutes; c__Clostridia; o__Clostridiales; f__Lachnospiraceae; g__; s__ |
| denovo923 | -0.100682249 | 4.38E-06 | 0.000109569 | k__Bacteria; p__Actinobacteria; c__Coriobacteriia; o__Coriobacteriales; f__Coriobacteriaceae; g__Atopobium; s__ |
| denovo808 | 0.100467928 | 5.25E-06 | 0.00012821 | k__Bacteria; p__Firmicutes; c__Clostridia; o__Clostridiales; f__; g__; s__ |
| denovo9 | 0.09883635 | 6.01E-06 | 0.000143895 | k__Bacteria; p__Firmicutes; c__Clostridia; o__Clostridiales; f__; g__; s__ |
| denovo761 | 0.097770941 | 8.01E-06 | 0.000184139 | k__Bacteria; p__Firmicutes; c__Clostridia; o__Clostridiales; f__; g__; s__ |
| denovo288 | -0.097459621 | 9.84E-06 | 0.000219582 | k__Bacteria; p__Firmicutes; c__Clostridia; o__Clostridiales; f__Ruminococcaceae; g__; s__ |
| denovo180 | 0.09580756 | 1.10E-05 | 0.000241012 | k__Bacteria; p__Firmicutes; c__Clostridia; o__Clostridiales; f__; g__; s__ |
| denovo153 | -0.094703433 | 1.59E-05 | 0.000330755 | k__Bacteria; p__Firmicutes; c__Clostridia; o__Clostridiales; f__Lachnospiraceae; g__Dorea; s__ |
| denovo473 | 0.093899698 | 1.65E-05 | 0.000341699 | k__Bacteria; p__Firmicutes; c__Clostridia; o__Clostridiales; f__Ruminococcaceae; g__; s__ |
| denovo362 | -0.094604514 | 1.82E-05 | 0.000370991 | k__Bacteria; p__Firmicutes; c__Clostridia; o__Clostridiales; f__Lachnospiraceae; g__Dorea; s__ |
| denovo12 | 0.093536503 | 1.82E-05 | 0.000371516 | k__Bacteria; p__Firmicutes; c__Clostridia; o__Clostridiales; f__Ruminococcaceae; g__; s__ |
| denovo270 | 0.092480621 | 2.02E-05 | 0.000404154 | k__Bacteria; p__Firmicutes; c__Clostridia; o__Clostridiales; f__; g__; s__ |
| denovo328 | -0.093313439 | 2.18E-05 | 0.000431683 | k__Bacteria; p__Firmicutes; c__Clostridia; o__Clostridiales; f__Ruminococcaceae; g__Ruminococcus; s__ |
| denovo202 | 0.092929652 | 2.41E-05 | 0.000470341 | k__Bacteria; p__Firmicutes; c__Clostridia; o__Clostridiales; f__Lachnospiraceae |
| denovo1143 | 0.092966471 | 2.43E-05 | 0.000473285 | k__Bacteria; p__Firmicutes; c__Clostridia; o__Clostridiales; f__Lachnospiraceae; g__; s__ |
| denovo155 | 0.092084689 | 2.50E-05 | 0.000485875 | k__Bacteria; p__Firmicutes; c__Clostridia; o__Clostridiales; f__Ruminococcaceae; g__; s__ |
| denovo165 | -0.092257506 | 2.77E-05 | 0.000528809 | k__Bacteria; p__Firmicutes; c__Clostridia; o__Clostridiales; f__Lachnospiraceae; g__; s__ |
| denovo62 | 0.090542699 | 3.00E-05 | 0.00056588 | k__Bacteria; p__Firmicutes; c__Clostridia; o__Clostridiales; f__; g__; s__ |
| denovo7 | 0.091767026 | 3.05E-05 | 0.000573341 | k__Bacteria; p__Firmicutes; c__Clostridia; o__Clostridiales; f__Ruminococcaceae; g__Faecalibacterium; s__prausnitzii |
| denovo120 | -0.091839523 | 3.15E-05 | 0.000589481 | k__Bacteria; p__Firmicutes; c__Clostridia; o__Clostridiales; f__Ruminococcaceae; g__Ruminococcus; s__ |
| denovo618 | -0.091604735 | 3.33E-05 | 0.000618675 | k__Bacteria; p__Firmicutes; c__Clostridia; o__Clostridiales; f__[Mogibacteriaceae]; g__; s__ |
| denovo448 | 0.089916818 | 3.97E-05 | 0.000716882 | k__Bacteria; p__Firmicutes; c__Clostridia; o__Clostridiales; f__Ruminococcaceae; g__; s__ |
| denovo256 | 0.089794359 | 4.17E-05 | 0.000747429 | k__Bacteria; p__Proteobacteria; c__Betaproteobacteria; o__Burkholderiales; f__Oxalobacteraceae; g__Oxalobacter; s__formigenes |
| denovo450 | 0.089689698 | 4.26E-05 | 0.00076005 | k__Bacteria; p__Firmicutes; c__Clostridia; o__Clostridiales; f__Ruminococcaceae; g__; s__ |
| denovo826 | -0.09002112 | 4.55E-05 | 0.000803615 | k__Bacteria; p__Firmicutes; c__Clostridia; o__Clostridiales; f__Eubacteriaceae; g__Anaerofustis; s__ |
| denovo546 | 0.087605109 | 6.81E-05 | 0.001128038 | k__Bacteria; p__Firmicutes; c__Clostridia; o__Clostridiales; f__; g__; s__ |
| denovo135 | 0.087265695 | 7.09E-05 | 0.001165528 | k__Bacteria; p__Firmicutes; c__Clostridia; o__Clostridiales; f__; g__; s__ |
| denovo283 | 0.086874199 | 7.93E-05 | 0.001279916 | k__Bacteria; p__Firmicutes; c__Clostridia; o__Clostridiales; f__Ruminococcaceae; g__Oscillospira; s__ |
| denovo1581 | 0.08691698 | 8.18E-05 | 0.001313967 | k__Bacteria; p__Firmicutes; c__Clostridia; o__Clostridiales; f__Ruminococcaceae; g__Oscillospira; s__ |
| denovo39 | 0.086315404 | 8.65E-05 | 0.001376967 | k__Bacteria; p__Proteobacteria; c__Gammaproteobacteria; o__Pasteurellales; f__Pasteurellaceae; g__Haemophilus |
| denovo295 | -0.085828937 | 9.66E-05 | 0.001509587 | k__Bacteria; p__Actinobacteria; c__Coriobacteriia; o__Coriobacteriales; f__Coriobacteriaceae; g__Eggerthella; s__lenta |
| denovo1014 | 0.085764745 | 0.000100556 | 0.001561252 | k__Bacteria; p__Firmicutes; c__Clostridia; o__Clostridiales; f__Lachnospiraceae; g__; s__ |
| denovo246 | 0.084613355 | 0.000114808 | 0.001743533 | k__Bacteria; p__Firmicutes; c__Clostridia; o__Clostridiales; f__Ruminococcaceae; g__; s__ |
| denovo438 | 0.083834524 | 0.000118185 | 0.001786117 | k__Bacteria; p__Firmicutes; c__Clostridia; o__Clostridiales; f__Ruminococcaceae; g__; s__ |
| denovo632 | -0.083457664 | 0.0001334 | 0.001975398 | k__Bacteria; p__Firmicutes; c__Bacilli; o__Lactobacillales; f__Streptococcaceae; g__Streptococcus; s__anginosus |
| denovo43 | 0.083751282 | 0.00014057 | 0.002063239 | k__Bacteria; p__Firmicutes; c__Clostridia; o__Clostridiales; f__Lachnospiraceae; g__Coprococcus |
| denovo52 | 0.082771021 | 0.000149744 | 0.002174464 | k__Bacteria; p__Firmicutes; c__Clostridia; o__Clostridiales; f__Clostridiaceae; g__Clostridium; s__ |
| denovo47 | 0.082755031 | 0.000154866 | 0.00223602 | k__Bacteria; p__Firmicutes; c__Clostridia; o__Clostridiales; f__Ruminococcaceae; g__; s__ |
| denovo193 | -0.083118788 | 0.000158282 | 0.002276865 | k__Bacteria; p__Firmicutes; c__Erysipelotrichi; o__Erysipelotrichales; f__Erysipelotrichaceae; g__; s__ |
| denovo258 | 0.082913873 | 0.000164344 | 0.002348969 | k__Bacteria; p__Firmicutes; c__Clostridia; o__Clostridiales; f__Ruminococcaceae; g__; s__ |
| denovo628 | -0.082549717 | 0.000179946 | 0.002532363 | k__Bacteria; p__Firmicutes; c__Bacilli; o__Gemellales; f__Gemellaceae; g__; s__ |
| denovo136 | 0.081513535 | 0.000196775 | 0.002726952 | k__Bacteria; p__Firmicutes; c__Clostridia; o__Clostridiales; f__; g__; s__ |
| denovo501 | 0.081409846 | 0.000198147 | 0.002742684 | k__Bacteria; p__Firmicutes; c__Clostridia; o__Clostridiales; f__[Mogibacteriaceae]; g__; s__ |
| denovo213 | -0.081451903 | 0.000201957 | 0.002786243 | k__Bacteria; p__Firmicutes; c__Clostridia; o__Clostridiales; f__Ruminococcaceae; g__Ruminococcus; s__ |
| denovo936 | 0.081191059 | 0.000207007 | 0.002843763 | k__Bacteria; p__Firmicutes; c__Clostridia; o__Clostridiales; f__Ruminococcaceae; g__Oscillospira; s__ |
| denovo1263 | -0.081445084 | 0.000215196 | 0.00293647 | k__Bacteria; p__Firmicutes; c__Clostridia; o__Clostridiales; f__Lachnospiraceae |
| denovo459 | 0.080237578 | 0.000271856 | 0.003561461 | k__Bacteria; p__Firmicutes; c__Clostridia; o__Clostridiales; f__Ruminococcaceae; g__; s__ |
| denovo36 | 0.079057635 | 0.000292056 | 0.003778144 | k__Bacteria; p__Firmicutes; c__Clostridia; o__Clostridiales; f__; g__; s__ |
| denovo770 | 0.07888613 | 0.000315252 | 0.004023491 | k__Bacteria; p__Firmicutes; c__Clostridia; o__Clostridiales; f__Ruminococcaceae |
| denovo561 | -0.079361245 | 0.000324425 | 0.004119558 | k__Bacteria; p__Firmicutes; c__Clostridia; o__Clostridiales; f__Ruminococcaceae; g__; s__ |
| denovo190 | -0.079044336 | 0.000338637 | 0.004267382 | k__Bacteria; p__Firmicutes; c__Clostridia; o__Clostridiales; f__Ruminococcaceae; g__Oscillospira; s__ |
| denovo84 | 0.077892916 | 0.000395735 | 0.004849855 | k__Bacteria; p__Proteobacteria; c__Betaproteobacteria; o__Burkholderiales; f__Alcaligenaceae; g__Sutterella; s__ |
| denovo1314 | 0.077776188 | 0.000400556 | 0.004898267 | k__Bacteria; p__Firmicutes; c__Clostridia; o__Clostridiales; f__; g__; s__ |
| denovo1264 | 0.07776968 | 0.000405541 | 0.004948198 | k__Bacteria; p__Firmicutes; c__Clostridia; o__Clostridiales; f__Ruminococcaceae; g__; s__ |
| denovo137 | 0.077833365 | 0.000410362 | 0.004996379 | k__Bacteria; p__Firmicutes; c__Clostridia; o__Clostridiales; f__; g__; s__ |
| denovo4 | -0.077406289 | 0.000422807 | 0.005120243 | k__Bacteria; p__Proteobacteria; c__Gammaproteobacteria; o__Enterobacteriales; f__Enterobacteriaceae; g__; s__ |
| denovo374 | 0.077180526 | 0.000437438 | 0.005264951 | k__Bacteria; p__Firmicutes; c__Clostridia; o__Clostridiales; f__Ruminococcaceae; g__; s__ |
| denovo1127 | 0.076375543 | 0.000508829 | 0.005958099 | k__Bacteria; p__Firmicutes; c__Clostridia; o__Clostridiales; f__Lachnospiraceae |
| denovo1050 | 0.076698094 | 0.00051191 | 0.005987563 | k__Bacteria; p__Firmicutes; c__Clostridia; o__Clostridiales; f__Ruminococcaceae; g__Faecalibacterium; s__prausnitzii |
| denovo430 | 0.075475748 | 0.000563022 | 0.00647136 | k__Bacteria; p__Firmicutes; c__Clostridia; o__Clostridiales; f__Clostridiaceae; g__; s__ |
| denovo814 | -0.07615921 | 0.00056558 | 0.006495341 | k__Bacteria; p__Firmicutes; c__Clostridia; o__Clostridiales; f__Ruminococcaceae; g__Ruminococcus; s__ |
| denovo594 | 0.07541348 | 0.000587683 | 0.006701608 | k__Bacteria; p__Firmicutes; c__Clostridia; o__Clostridiales; f__Ruminococcaceae; g__; s__ |
| denovo461 | -0.074382577 | 0.000695788 | 0.00768915 | k__Bacteria; p__Firmicutes; c__Bacilli; o__Lactobacillales; f__Lactobacillaceae; g__Lactobacillus; s__zeae |
| denovo49 | 0.074340332 | 0.000699059 | 0.007718522 | k__Bacteria; p__Firmicutes; c__Clostridia; o__Clostridiales; f__Ruminococcaceae; g__; s__ |
| denovo82 | 0.074131816 | 0.000760914 | 0.008268746 | k__Bacteria; p__Bacteroidetes; c__Bacteroidia; o__Bacteroidales; f__[Odoribacteraceae]; g__Odoribacter; s__ |
| denovo68 | 0.073679787 | 0.000809888 | 0.008697838 | k__Bacteria; p__Firmicutes; c__Clostridia; o__Clostridiales; f__; g__; s__ |
| denovo372 | -0.073799267 | 0.000818651 | 0.008774033 | k__Bacteria; p__Fusobacteria; c__Fusobacteriia; o__Fusobacteriales; f__Fusobacteriaceae; g__Fusobacterium; s__ |
| denovo272 | -0.073525425 | 0.000823006 | 0.008811844 | k__Bacteria; p__Firmicutes; c__Clostridia; o__Clostridiales; f__Lachnospiraceae; g__; s__ |
| denovo292 | 0.073588959 | 0.000849623 | 0.009042004 | k__Bacteria; p__Actinobacteria; c__Coriobacteriia; o__Coriobacteriales; f__Coriobacteriaceae; g__; s__ |
| denovo26 | 0.073434395 | 0.000855969 | 0.009096659 | k__Bacteria; p__Firmicutes; c__Clostridia; o__Clostridiales; f__Lachnospiraceae; g__; s__ |
| denovo1588 | 0.073220208 | 0.000903377 | 0.009502295 | k__Bacteria; p__Firmicutes; c__Clostridia; o__Clostridiales; f__Lachnospiraceae; g__; s__ |
| denovo1524 | 0.072208264 | 0.000954994 | 0.009938872 | k__Bacteria; p__Firmicutes; c__Clostridia; o__Clostridiales; f__; g__; s__ |
| denovo397 | 0.072226811 | 0.000959639 | 0.009977907 | k__Bacteria; p__Firmicutes; c__Clostridia; o__Clostridiales; f__Ruminococcaceae; g__; s__ |
| denovo115 | 0.072184639 | 0.001042866 | 0.010670775 | k__Bacteria; p__Tenericutes; c__Mollicutes; o__RF39; f__; g__; s__ |
| denovo572 | 0.072116057 | 0.001083593 | 0.011005469 | k__Bacteria; p__Firmicutes; c__Clostridia; o__Clostridiales; f__Ruminococcaceae; g__; s__ |
| denovo207 | 0.070931609 | 0.001196639 | 0.011920669 | k__Bacteria; p__Firmicutes; c__Clostridia; o__Clostridiales; f__Ruminococcaceae; g__; s__ |
| denovo1315 | 0.071466938 | 0.001213137 | 0.012052623 | k__Bacteria; p__Firmicutes; c__Clostridia; o__Clostridiales; f__Lachnospiraceae; g__; s__ |
| denovo355 | 0.070543953 | 0.001329058 | 0.012969061 | k__Bacteria; p__Firmicutes; c__Clostridia; o__Clostridiales; f__; g__; s__ |
| denovo205 | -0.070580209 | 0.001354996 | 0.013171668 | k__Bacteria; p__Firmicutes; c__Clostridia; o__Clostridiales; f__[Mogibacteriaceae]; g__; s__ |
| denovo41 | 0.070449752 | 0.001373702 | 0.01331725 | k__Bacteria; p__Tenericutes; c__Mollicutes; o__RF39; f__; g__; s__ |
| denovo1764 | 0.070101821 | 0.001388581 | 0.013432727 | k__Bacteria; p__Firmicutes; c__Clostridia; o__Clostridiales; f__; g__; s__ |
| denovo465 | -0.070377898 | 0.001435437 | 0.013794618 | k__Bacteria; p__Firmicutes; c__Clostridia; o__Clostridiales; f__Ruminococcaceae; g__Anaerotruncus; s__ |
| denovo668 | -0.070195569 | 0.001480739 | 0.014142014 | k__Bacteria; p__Firmicutes; c__Clostridia; o__Clostridiales; f__Ruminococcaceae; g__; s__ |
| denovo828 | 0.069947491 | 0.001530767 | 0.014522892 | k__Bacteria; p__Firmicutes; c__Clostridia; o__Clostridiales; f__Lachnospiraceae; g__; s__ |
| denovo25 | -0.069716277 | 0.00156378 | 0.014772702 | k__Bacteria; p__Firmicutes; c__Clostridia; o__Clostridiales; f__Lachnospiraceae; g__Blautia; s__ |
| denovo147 | 0.06974024 | 0.001570786 | 0.014825555 | k__Bacteria; p__Tenericutes; c__Mollicutes; o__RF39; f__; g__; s__ |
| denovo122 | 0.069756057 | 0.001587135 | 0.014948703 | k__Bacteria; p__Firmicutes; c__Clostridia; o__Clostridiales; f__Ruminococcaceae; g__; s__ |
| denovo587 | -0.069644774 | 0.001624392 | 0.015228275 | k__Bacteria; p__Firmicutes; c__Clostridia; o__Clostridiales; f__Ruminococcaceae; g__; s__ |
| denovo64 | 0.068788384 | 0.001661077 | 0.015502128 | k__Bacteria; p__Firmicutes; c__Clostridia; o__Clostridiales |
| denovo112 | 0.069081851 | 0.001669488 | 0.015564727 | k__Bacteria; p__Proteobacteria; c__Alphaproteobacteria; o__RF32; f__; g__; s__ |
| denovo323 | 0.067671665 | 0.001932528 | 0.017487984 | k__Bacteria; p__Firmicutes; c__Clostridia; o__Clostridiales; f__Ruminococcaceae; g__; s__ |
| denovo959 | 0.067250412 | 0.002228069 | 0.019577848 | k__Bacteria; p__Firmicutes; c__Clostridia; o__Clostridiales; f__Ruminococcaceae; g__; s__ |
| denovo433 | 0.066848794 | 0.002323326 | 0.020237226 | k__Bacteria; p__Firmicutes; c__Clostridia; o__Clostridiales; f__Christensenellaceae; g__; s__ |
| denovo1126 | -0.066393656 | 0.002603874 | 0.022143116 | k__Bacteria; p__Firmicutes; c__Clostridia; o__Clostridiales |
| denovo254 | 0.065594071 | 0.00272942 | 0.022979816 | k__Bacteria; p__Firmicutes; c__Clostridia; o__Clostridiales; f__; g__; s__ |
| denovo331 | 0.065797853 | 0.002761049 | 0.023189124 | k__Bacteria; p__Firmicutes; c__Clostridia; o__Clostridiales; f__Clostridiaceae; g__; s__ |
| denovo410 | 0.064826798 | 0.003222127 | 0.026177788 | k__Bacteria; p__Firmicutes; c__Clostridia; o__Clostridiales; f__Ruminococcaceae; g__; s__ |
| denovo1137 | 0.06439008 | 0.003478847 | 0.027795486 | k__Bacteria; p__Firmicutes; c__Clostridia; o__Clostridiales; f__Lachnospiraceae; g__; s__ |
| denovo412 | -0.064223222 | 0.003489785 | 0.027863733 | k__Bacteria; p__Firmicutes; c__Clostridia; o__Clostridiales; f__[Mogibacteriaceae]; g__; s__ |
| denovo441 | 0.064278246 | 0.003567615 | 0.028347821 | k__Bacteria; p__Firmicutes; c__Clostridia; o__Clostridiales; f__Ruminococcaceae; g__; s__ |
| denovo60 | -0.06325304 | 0.003743275 | 0.029430765 | k__Bacteria; p__Firmicutes; c__Bacilli; o__Lactobacillales; f__Streptococcaceae; g__Streptococcus; s__ |
| denovo329 | -0.063712368 | 0.00374364 | 0.029433003 | k__Bacteria; p__Firmicutes; c__Clostridia; o__Clostridiales; f__Lachnospiraceae; g__Dorea; s__ |
| denovo956 | 0.063182108 | 0.003988001 | 0.030918318 | k__Bacteria; p__Firmicutes; c__Clostridia; o__Clostridiales; f__Ruminococcaceae; g__; s__ |
| denovo1398 | 0.063402809 | 0.003998495 | 0.030981576 | k__Bacteria; p__Firmicutes; c__Clostridia; o__Clostridiales; f__Lachnospiraceae; g__Blautia; s__ |
| denovo1129 | 0.063191943 | 0.004023735 | 0.031133551 | k__Bacteria; p__Firmicutes; c__Clostridia; o__Clostridiales |
| denovo1332 | 0.063144641 | 0.004166341 | 0.031987684 | k__Bacteria; p__Firmicutes; c__Clostridia; o__Clostridiales; f__Ruminococcaceae; g__; s__ |
| denovo154 | 0.062825714 | 0.004251583 | 0.03249465 | k__Bacteria; p__Firmicutes; c__Clostridia; o__Clostridiales; f__; g__; s__ |
| denovo530 | -0.062531021 | 0.004621108 | 0.034662878 | k__Bacteria; p__Proteobacteria; c__Betaproteobacteria; o__Burkholderiales; f__Oxalobacteraceae; g__Ralstonia; s__ |
| denovo367 | -0.061921207 | 0.005022126 | 0.036965406 | k__Bacteria; p__Firmicutes; c__Clostridia; o__Clostridiales; f__Lachnospiraceae; g__; s__ |
| denovo211 | 0.06147569 | 0.005216283 | 0.038062699 | Unassigned |
| denovo182 | -0.061268287 | 0.005399332 | 0.039087353 | k__Bacteria; p__Firmicutes; c__Clostridia; o__Clostridiales; f__Ruminococcaceae; g__Ruminococcus; s__ |
| denovo494 | -0.060717568 | 0.005991936 | 0.04234317 | k__Bacteria; p__Bacteroidetes; c__Bacteroidia; o__Bacteroidales; f__Porphyromonadaceae; g__Porphyromonas; s__ |
| denovo1390 | 0.060287818 | 0.006050951 | 0.042662545 | k__Bacteria; p__Firmicutes; c__Clostridia; o__Clostridiales; f__Ruminococcaceae; g__; s__ |
| denovo1223 | 0.060546156 | 0.006128996 | 0.043083618 | k__Bacteria; p__Firmicutes; c__Clostridia; o__Clostridiales; f__Ruminococcaceae; g__; s__ |
| denovo311 | 0.059877505 | 0.006153682 | 0.043216501 | k__Bacteria; p__Firmicutes; c__Clostridia; o__Clostridiales; f__Ruminococcaceae; g__; s__ |
| denovo1335 | 0.059923037 | 0.006308224 | 0.044045143 | k__Bacteria; p__Firmicutes; c__Clostridia; o__Clostridiales; f__Clostridiaceae |
| denovo1015 | 0.059554861 | 0.006635298 | 0.045780946 | k__Bacteria; p__Firmicutes; c__Clostridia; o__Clostridiales; f__; g__; s__ |
| denovo97 | -0.059547753 | 0.006927305 | 0.047310951 | k__Bacteria; p__Firmicutes; c__Clostridia; o__Clostridiales; f__Lachnospiraceae; g__Dorea; s__formicigenerans |
| denovo214 | 0.059391779 | 0.007169774 | 0.048567951 | k__Bacteria; p__Cyanobacteria; c__Chloroplast; o__Streptophyta; f__; g__; s__ |
| denovo238 | 0.058586698 | 0.007321161 | 0.049346801 | k__Bacteria; p__Firmicutes; c__Clostridia; o__Clostridiales; f__; g__; s__ |

**Additional file 1: Table S8.** Results of mixed effects models of OTU associations collapsed to genus level with the Healthy Eating Index (see methods). Number of significant associations = 16

| genus | Beta | Nom_P | FDR.P |
| --- | --- | --- | --- |
| k__Bacteria.p__Actinobacteria.c__Actinobacteria.o__Actinomycetales.f__Actinomycetaceae.g__Actinomyces | -0.130042322 | 2.69E-09 | 1.69E-05 |
| k__Bacteria.p__Firmicutes.c__Clostridia.o__Clostridiales.f__Lachnospiraceae.g__.Ruminococcus. | -0.121633236 | 2.69E-08 | 4.09E-05 |
| k__Bacteria.p__Firmicutes.c__Clostridia.o__Clostridiales.f__Lachnospiraceae.g__Lachnospira | 0.106420859 | 1.12E-06 | 0.000879615 |
| k__Bacteria.p__Firmicutes.c__Clostridia.o__Clostridiales.f__Peptococcaceae.g__ | 0.100164075 | 4.74E-06 | 0.002233129 |
| k__Bacteria.p__Firmicutes.c__Clostridia.o__Clostridiales.f__Lachnospiraceae.g__Dorea | -0.098917726 | 6.40E-06 | 0.00273906 |
| k__Bacteria.p__Actinobacteria.c__Coriobacteriia.o__Coriobacteriales.f__Coriobacteriaceae.g__Atopobium | -0.098281677 | 7.27E-06 | 0.002988987 |
| k__Bacteria.p__Tenericutes.c__Mollicutes.o__Anaeroplasmatales.f__Anaeroplasmataceae.g__ | 0.092051962 | 2.60E-05 | 0.007200331 |
| k__Bacteria.p__Firmicutes.c__Clostridia.o__Clostridiales.f__Lachnospiraceae.g__Blautia | -0.087930074 | 6.44E-05 | 0.013372314 |
| k__Bacteria.p__Proteobacteria.c__Betaproteobacteria.o__Burkholderiales.f__Oxalobacteraceae.g__Oxalobacter | 0.087249229 | 6.84E-05 | 0.013928541 |
| k__Bacteria.p__Fusobacteria.c__Fusobacteriia.o__Fusobacteriales.f__Fusobacteriaceae.g__Fusobacterium | -0.087371978 | 7.21E-05 | 0.01444008 |
| k__Bacteria.p__Tenericutes.c__RF3.o__ML615J.28.f__.g__ | 0.086001147 | 7.94E-05 | 0.015414848 |
| k__Bacteria.p__Actinobacteria.c__Coriobacteriia.o__Coriobacteriales.f__Coriobacteriaceae.g__Eggerthella | -0.084570312 | 0.000120822 | 0.020519581 |
| k__Bacteria.p__Firmicutes.c__Bacilli.o__Gemellales.f__Gemellaceae.g__ | -0.081657574 | 0.000210443 | 0.029986691 |
| k__Bacteria.p__Tenericutes.c__Mollicutes.o__RF39.f__.g__ | 0.079909333 | 0.000235144 | 0.032363052 |
| k__Bacteria.p__Firmicutes.c__Clostridia.o__Clostridiales.f__Eubacteriaceae.g__Anaerofustis | -0.07954148 | 0.000299955 | 0.038277947 |
| k__Bacteria.p__Lentisphaerae.c__.Lentisphaeria..o__Victivallales.f__Victivallaceae.g__ | 0.077071513 | 0.000423447 | 0.048623712 |

**Additional file 1: Table S9.** Results of mixed effects models of OTU associations collapsed to phylum level with the Healthy Eating Index (see methods). Only FDR significant results listed, Number of significant results = 4

| phylum | Beta | Nom_P | FDR.P |
| --- | --- | --- | --- |
| k__Bacteria.p__Tenericutes | 0.092068817 | 2.14E-05 | 0.001065091 |
| k__Bacteria.p__Fusobacteria | -0.078481719 | 0.000372451 | 0.010079227 |
| k__Bacteria.p__Lentisphaerae | 0.077212282 | 0.000413103 | 0.010985332 |
| k__Bacteria.p__Chlorobi | -0.066368347 | 0.002441926 | 0.048651073 |

**Additional file 1: Table S10.** FDR significant OTU associations with the MDS, Number of significant associations = 107

| **OTU** | **Beta** | **Nom_P** | **FDR.P** | **Assigned.Taxonomy** |
| --- | --- | --- | --- | --- |
| denovo27 | -0.129533305 | 3.72E-09 | 3.12E-06 | k__Bacteria; p__Firmicutes; c__Clostridia; o__Clostridiales; f__Lachnospiraceae; g__[Ruminococcus]; s__gnavus |
| denovo277 | 0.154666268 | 2.09E-12 | 3.12E-06 | k__Bacteria; p__Firmicutes; c__Clostridia; o__Clostridiales; f__; g__; s__ |
| denovo55 | -0.123683559 | 1.79E-08 | 4.79E-06 | k__Bacteria; p__Firmicutes; c__Clostridia; o__Clostridiales; f__Lachnospiraceae; g__[Ruminococcus]; s__ |
| denovo57 | 0.122796532 | 2.03E-08 | 5.26E-06 | k__Bacteria; p__Firmicutes; c__Clostridia; o__Clostridiales; f__Lachnospiraceae; g__Lachnospira; s__ |
| denovo129 | 0.114934968 | 2.41E-07 | 3.17E-05 | k__Bacteria; p__Firmicutes; c__Clostridia; o__Clostridiales; f__Lachnospiraceae; g__Lachnospira; s__ |
| denovo102 | 0.11355667 | 2.87E-07 | 3.58E-05 | k__Bacteria; p__Firmicutes; c__Clostridia; o__Clostridiales; f__Ruminococcaceae; g__Oscillospira; s__ |
| denovo428 | 0.106935705 | 1.18E-06 | 9.61E-05 | k__Bacteria; p__Firmicutes; c__Clostridia; o__Clostridiales; f__Peptococcaceae; g__; s__ |
| denovo151 | 0.105399722 | 1.48E-06 | 0.000112807 | k__Bacteria; p__Firmicutes; c__Clostridia; o__Clostridiales; f__; g__; s__ |
| denovo270 | 0.101356433 | 3.29E-06 | 0.000197912 | k__Bacteria; p__Firmicutes; c__Clostridia; o__Clostridiales; f__; g__; s__ |
| denovo445 | 0.102091101 | 3.90E-06 | 0.00022343 | k__Bacteria; p__Firmicutes; c__Clostridia; o__Clostridiales; f__Lachnospiraceae; g__Coprococcus; s__ |
| denovo467 | -0.09982737 | 6.04E-06 | 0.000305809 | k__Bacteria; p__Actinobacteria; c__Actinobacteria; o__Actinomycetales; f__Actinomycetaceae; g__Actinomyces; s__ |
| denovo365 | 0.099639458 | 6.20E-06 | 0.000311392 | k__Bacteria; p__Firmicutes; c__Clostridia; o__Clostridiales; f__[Mogibacteriaceae]; g__; s__ |
| denovo344 | 0.09957321 | 6.71E-06 | 0.000329945 | k__Bacteria; p__Firmicutes; c__Clostridia; o__Clostridiales; f__Ruminococcaceae; g__; s__ |
| denovo604 | 0.100428854 | 7.98E-06 | 0.000373873 | k__Bacteria; p__Firmicutes; c__Clostridia; o__Clostridiales; f__; g__; s__ |
| denovo753 | -0.096780142 | 1.06E-05 | 0.000460625 | k__Bacteria; p__Firmicutes; c__Clostridia; o__Clostridiales; f__Lachnospiraceae; g__; s__ |
| denovo552 | 0.095654594 | 1.31E-05 | 0.000537739 | k__Bacteria; p__Firmicutes; c__Clostridia; o__Clostridiales; f__Christensenellaceae; g__; s__ |
| denovo313 | 0.095896952 | 1.67E-05 | 0.00064261 | k__Bacteria; p__Firmicutes; c__Clostridia; o__Clostridiales; f__Lachnospiraceae; g__; s__ |
| denovo375 | 0.095055597 | 1.73E-05 | 0.000659122 | k__Bacteria; p__Firmicutes; c__Clostridia; o__Clostridiales; f__; g__; s__ |
| denovo256 | 0.094612334 | 1.73E-05 | 0.000659167 | k__Bacteria; p__Proteobacteria; c__Betaproteobacteria; o__Burkholderiales; f__Oxalobacteraceae; g__Oxalobacter; s__formigenes |
| denovo284 | 0.096140507 | 1.91E-05 | 0.000710215 | k__Bacteria; p__Firmicutes; c__Clostridia; o__Clostridiales; f__Ruminococcaceae; g__; s__ |
| denovo88 | -0.094337792 | 2.01E-05 | 0.000738673 | k__Bacteria; p__Firmicutes; c__Clostridia; o__Clostridiales; f__Ruminococcaceae; g__; s__ |
| denovo383 | 0.094447058 | 2.04E-05 | 0.000747004 | k__Bacteria; p__Firmicutes; c__Clostridia; o__Clostridiales; f__Lachnospiraceae; g__; s__ |
| denovo1014 | 0.09568572 | 2.23E-05 | 0.000796087 | k__Bacteria; p__Firmicutes; c__Clostridia; o__Clostridiales; f__Lachnospiraceae; g__; s__ |
| denovo469 | 0.093791874 | 2.91E-05 | 0.000973493 | k__Bacteria; p__Firmicutes; c__Clostridia; o__Clostridiales; f__Ruminococcaceae; g__Faecalibacterium; s__prausnitzii |
| denovo123 | 0.091267491 | 3.15E-05 | 0.001033061 | k__Bacteria; p__Firmicutes; c__Clostridia; o__Clostridiales; f__Ruminococcaceae; g__; s__ |
| denovo336 | 0.091023316 | 3.39E-05 | 0.001092018 | k__Bacteria; p__Firmicutes; c__Clostridia; o__Clostridiales; f__Ruminococcaceae; g__Oscillospira; s__ |
| denovo110 | 0.091085815 | 3.87E-05 | 0.001206221 | k__Bacteria; p__Firmicutes; c__Clostridia; o__Clostridiales; f__Ruminococcaceae; g__; s__ |
| denovo512 | -0.090522482 | 5.06E-05 | 0.001475511 | k__Bacteria; p__Firmicutes; c__Clostridia; o__Clostridiales; f__[Mogibacteriaceae]; g__; s__ |
| denovo76 | 0.089632309 | 5.32E-05 | 0.001531344 | k__Bacteria; p__Firmicutes; c__Clostridia; o__Clostridiales; f__Ruminococcaceae; g__; s__ |
| denovo1160 | -0.089054967 | 5.87E-05 | 0.001649602 | k__Bacteria; p__Firmicutes; c__Clostridia; o__Clostridiales; f__; g__; s__ |
| denovo180 | 0.087899602 | 6.73E-05 | 0.001830104 | k__Bacteria; p__Firmicutes; c__Clostridia; o__Clostridiales; f__; g__; s__ |
| denovo13 | -0.087362522 | 7.13E-05 | 0.001910807 | k__Bacteria; p__Firmicutes; c__Clostridia; o__Clostridiales; f__Lachnospiraceae; g__Blautia; s__ |
| denovo526 | 0.087348768 | 7.90E-05 | 0.002064954 | k__Bacteria; p__Firmicutes; c__Clostridia; o__Clostridiales; f__Ruminococcaceae; g__Oscillospira; s__ |
| denovo155 | 0.086484686 | 8.22E-05 | 0.002128072 | k__Bacteria; p__Firmicutes; c__Clostridia; o__Clostridiales; f__Ruminococcaceae; g__; s__ |
| denovo450 | 0.085891621 | 9.61E-05 | 0.002396073 | k__Bacteria; p__Firmicutes; c__Clostridia; o__Clostridiales; f__Ruminococcaceae; g__; s__ |
| denovo441 | 0.088884403 | 0.00010579 | 0.002575758 | k__Bacteria; p__Firmicutes; c__Clostridia; o__Clostridiales; f__Ruminococcaceae; g__; s__ |
| denovo448 | 0.084943084 | 0.000112351 | 0.002695538 | k__Bacteria; p__Firmicutes; c__Clostridia; o__Clostridiales; f__Ruminococcaceae; g__; s__ |
| denovo1581 | 0.08715684 | 0.000120712 | 0.00284569 | k__Bacteria; p__Firmicutes; c__Clostridia; o__Clostridiales; f__Ruminococcaceae; g__Oscillospira; s__ |
| denovo272 | -0.084554026 | 0.000128276 | 0.002979275 | k__Bacteria; p__Firmicutes; c__Clostridia; o__Clostridiales; f__Lachnospiraceae; g__; s__ |
| denovo958 | 0.081718803 | 0.000185538 | 0.00393431 | k__Bacteria; p__Firmicutes; c__Clostridia; o__Clostridiales; f__; g__; s__ |
| denovo1898 | 0.0844759 | 0.000188776 | 0.003985808 | k__Bacteria; p__Firmicutes; c__Clostridia; o__Clostridiales; f__Lachnospiraceae; g__; s__ |
| denovo72 | 0.08152839 | 0.00019208 | 0.004038141 | k__Bacteria; p__Firmicutes; c__Clostridia; o__Clostridiales; f__; g__; s__ |
| denovo1116 | 0.081356982 | 0.000193013 | 0.00405287 | k__Bacteria; p__Firmicutes; c__Clostridia; o__Clostridiales; f__; g__; s__ |
| denovo288 | -0.085999899 | 0.000195791 | 0.004096625 | k__Bacteria; p__Firmicutes; c__Clostridia; o__Clostridiales; f__Ruminococcaceae; g__; s__ |
| denovo109 | 0.082138733 | 0.000199251 | 0.004150902 | k__Bacteria; p__Firmicutes; c__Clostridia; o__Clostridiales; f__; g__; s__ |
| denovo276 | 0.081761307 | 0.000212055 | 0.004349635 | k__Bacteria; p__Firmicutes; c__Clostridia; o__Clostridiales; f__Ruminococcaceae; g__Oscillospira; s__ |
| denovo154 | 0.080005004 | 0.000288695 | 0.005480212 | k__Bacteria; p__Firmicutes; c__Clostridia; o__Clostridiales; f__; g__; s__ |
| denovo79 | 0.080191169 | 0.000293915 | 0.005554059 | k__Bacteria; p__Firmicutes; c__Clostridia; o__Clostridiales; f__Ruminococcaceae; g__; s__ |
| denovo1524 | 0.079829949 | 0.000326681 | 0.006009853 | k__Bacteria; p__Firmicutes; c__Clostridia; o__Clostridiales; f__; g__; s__ |
| denovo1137 | 0.078509381 | 0.000504932 | 0.008304491 | k__Bacteria; p__Firmicutes; c__Clostridia; o__Clostridiales; f__Lachnospiraceae; g__; s__ |
| denovo974 | -0.077788395 | 0.000526111 | 0.008560675 | k__Bacteria; p__Firmicutes; c__Clostridia; o__Clostridiales; f__Lachnospiraceae; g__; s__ |
| denovo473 | 0.075194294 | 0.000604156 | 0.009480993 | k__Bacteria; p__Firmicutes; c__Clostridia; o__Clostridiales; f__Ruminococcaceae; g__; s__ |
| denovo202 | 0.077088329 | 0.000621124 | 0.00967654 | k__Bacteria; p__Firmicutes; c__Clostridia; o__Clostridiales; f__Lachnospiraceae |
| denovo461 | -0.075352572 | 0.000628554 | 0.009761699 | k__Bacteria; p__Firmicutes; c__Bacilli; o__Lactobacillales; f__Lactobacillaceae; g__Lactobacillus; s__zeae |
| denovo705 | -0.075467216 | 0.00063619 | 0.009848918 | k__Bacteria; p__Firmicutes; c__Clostridia; o__Clostridiales; f__Ruminococcaceae; g__Ruminococcus; s__ |
| denovo1588 | 0.075791714 | 0.000695267 | 0.010514065 | k__Bacteria; p__Firmicutes; c__Clostridia; o__Clostridiales; f__Lachnospiraceae; g__; s__ |
| denovo93 | 0.074979483 | 0.000707056 | 0.010644857 | k__Bacteria; p__Tenericutes; c__Mollicutes; o__RF39; f__; g__; s__ |
| denovo62 | 0.072995111 | 0.000820049 | 0.011868818 | k__Bacteria; p__Firmicutes; c__Clostridia; o__Clostridiales; f__; g__; s__ |
| denovo56 | 0.072626378 | 0.000923908 | 0.012951939 | k__Bacteria; p__Firmicutes; c__Clostridia; o__Clostridiales; f__Lachnospiraceae; g__; s__ |
| denovo84 | 0.073635144 | 0.000945511 | 0.013172751 | k__Bacteria; p__Proteobacteria; c__Betaproteobacteria; o__Burkholderiales; f__Alcaligenaceae; g__Sutterella; s__ |
| denovo1026 | -0.071969991 | 0.000992723 | 0.01365036 | k__Bacteria; p__Firmicutes; c__Clostridia; o__Clostridiales; f__Lachnospiraceae; g__[Ruminococcus]; s__gnavus |
| denovo136 | 0.072450526 | 0.000994687 | 0.01367008 | k__Bacteria; p__Firmicutes; c__Clostridia; o__Clostridiales; f__; g__; s__ |
| denovo374 | 0.075864712 | 0.001027533 | 0.013998336 | k__Bacteria; p__Firmicutes; c__Clostridia; o__Clostridiales; f__Ruminococcaceae; g__; s__ |
| denovo826 | -0.072871678 | 0.001182843 | 0.015511675 | k__Bacteria; p__Firmicutes; c__Clostridia; o__Clostridiales; f__Eubacteriaceae; g__Anaerofustis; s__ |
| denovo60 | -0.071034793 | 0.001191893 | 0.015598023 | k__Bacteria; p__Firmicutes; c__Bacilli; o__Lactobacillales; f__Streptococcaceae; g__Streptococcus; s__ |
| denovo97 | -0.071528182 | 0.001241405 | 0.016067157 | k__Bacteria; p__Firmicutes; c__Clostridia; o__Clostridiales; f__Lachnospiraceae; g__Dorea; s__formicigenerans |
| denovo33 | 0.072988111 | 0.001270109 | 0.016336648 | k__Bacteria; p__Firmicutes; c__Clostridia; o__Clostridiales; f__Ruminococcaceae; g__; s__ |
| denovo561 | -0.071484138 | 0.001274951 | 0.01638193 | k__Bacteria; p__Firmicutes; c__Clostridia; o__Clostridiales; f__Ruminococcaceae; g__; s__ |
| denovo295 | -0.071659681 | 0.001308537 | 0.01669469 | k__Bacteria; p__Actinobacteria; c__Coriobacteriia; o__Coriobacteriales; f__Coriobacteriaceae; g__Eggerthella; s__lenta |
| denovo59 | 0.070628043 | 0.001312717 | 0.016733456 | k__Bacteria; p__Firmicutes; c__Clostridia; o__Clostridiales; f__Ruminococcaceae; g__Oscillospira; s__ |
| denovo213 | -0.070759013 | 0.00131933 | 0.01679471 | k__Bacteria; p__Firmicutes; c__Clostridia; o__Clostridiales; f__Ruminococcaceae; g__Ruminococcus; s__ |
| denovo212 | 0.071680438 | 0.001381214 | 0.017363654 | k__Bacteria; p__Firmicutes; c__Clostridia; o__Clostridiales; f__Ruminococcaceae; g__; s__ |
| denovo430 | 0.06883889 | 0.001552616 | 0.018902368 | k__Bacteria; p__Firmicutes; c__Clostridia; o__Clostridiales; f__Clostridiaceae; g__; s__ |
| denovo299 | 0.068998809 | 0.001607606 | 0.019385386 | k__Bacteria; p__Firmicutes; c__Clostridia; o__Clostridiales; f__; g__; s__ |
| denovo691 | 0.068398114 | 0.001635082 | 0.019624924 | k__Bacteria; p__Firmicutes; c__Clostridia; o__Clostridiales; f__Dehalobacteriaceae; g__Dehalobacterium; s__ |
| denovo197 | 0.069027046 | 0.001649164 | 0.019747237 | k__Bacteria; p__Firmicutes; c__Clostridia; o__Clostridiales; f__Clostridiaceae; g__; s__ |
| denovo1764 | 0.069605287 | 0.001651893 | 0.01977091 | k__Bacteria; p__Firmicutes; c__Clostridia; o__Clostridiales; f__; g__; s__ |
| denovo120 | -0.071806373 | 0.001655636 | 0.01980335 | k__Bacteria; p__Firmicutes; c__Clostridia; o__Clostridiales; f__Ruminococcaceae; g__Ruminococcus; s__ |
| denovo546 | 0.070616355 | 0.001685633 | 0.020062605 | k__Bacteria; p__Firmicutes; c__Clostridia; o__Clostridiales; f__; g__; s__ |
| denovo43 | 0.069281211 | 0.001733598 | 0.020474368 | k__Bacteria; p__Firmicutes; c__Clostridia; o__Clostridiales; f__Lachnospiraceae; g__Coprococcus |
| denovo362 | -0.071279786 | 0.001831224 | 0.021302365 | k__Bacteria; p__Firmicutes; c__Clostridia; o__Clostridiales; f__Lachnospiraceae; g__Dorea; s__ |
| denovo1143 | 0.069508258 | 0.001902648 | 0.021899996 | k__Bacteria; p__Firmicutes; c__Clostridia; o__Clostridiales; f__Lachnospiraceae; g__; s__ |
| denovo1157 | 0.070952535 | 0.001950855 | 0.022299653 | k__Bacteria; p__Bacteroidetes; c__Bacteroidia; o__Bacteroidales; f__Bacteroidaceae; g__Bacteroides |
| denovo112 | 0.068041278 | 0.00206255 | 0.023214778 | k__Bacteria; p__Proteobacteria; c__Alphaproteobacteria; o__RF32; f__; g__; s__ |
| denovo1220 | 0.067760416 | 0.002076143 | 0.023325145 | k__Bacteria; p__Firmicutes; c__Clostridia; o__Clostridiales; f__; g__; s__ |
| denovo90 | 0.066396253 | 0.00252283 | 0.026843985 | k__Bacteria; p__Firmicutes; c__Clostridia; o__Clostridiales; f__Lachnospiraceae; g__Coprococcus; s__ |
| denovo49 | 0.066404848 | 0.002590032 | 0.027356923 | k__Bacteria; p__Firmicutes; c__Clostridia; o__Clostridiales; f__Ruminococcaceae; g__; s__ |
| denovo412 | -0.066577661 | 0.002785845 | 0.028829832 | k__Bacteria; p__Firmicutes; c__Clostridia; o__Clostridiales; f__[Mogibacteriaceae]; g__; s__ |
| denovo465 | -0.06594347 | 0.00296129 | 0.030123915 | k__Bacteria; p__Firmicutes; c__Clostridia; o__Clostridiales; f__Ruminococcaceae; g__Anaerotruncus; s__ |
| denovo1332 | 0.066363511 | 0.002968405 | 0.030175911 | k__Bacteria; p__Firmicutes; c__Clostridia; o__Clostridiales; f__Ruminococcaceae; g__; s__ |
| denovo147 | 0.067290047 | 0.003050313 | 0.030771914 | k__Bacteria; p__Tenericutes; c__Mollicutes; o__RF39; f__; g__; s__ |
| denovo278 | -0.066421068 | 0.003143419 | 0.031443726 | k__Bacteria; p__Firmicutes; c__Erysipelotrichi; o__Erysipelotrichales; f__Erysipelotrichaceae; g__[Eubacterium]; s__dolichum |
| denovo459 | 0.065190005 | 0.003260158 | 0.032277887 | k__Bacteria; p__Firmicutes; c__Clostridia; o__Clostridiales; f__Ruminococcaceae; g__; s__ |
| denovo936 | 0.064531385 | 0.003357575 | 0.032967296 | k__Bacteria; p__Firmicutes; c__Clostridia; o__Clostridiales; f__Ruminococcaceae; g__Oscillospira; s__ |
| denovo25 | -0.065164913 | 0.003556146 | 0.0343547 | k__Bacteria; p__Firmicutes; c__Clostridia; o__Clostridiales; f__Lachnospiraceae; g__Blautia; s__ |
| denovo923 | -0.063025727 | 0.004282475 | 0.039248569 | k__Bacteria; p__Actinobacteria; c__Coriobacteriia; o__Coriobacteriales; f__Coriobacteriaceae; g__Atopobium; s__ |
| denovo482 | 0.062910563 | 0.004296856 | 0.039342882 | k__Bacteria; p__Firmicutes; c__Clostridia; o__Clostridiales; f__; g__; s__ |
| denovo1432 | 0.064863494 | 0.004325053 | 0.039527538 | k__Bacteria; p__Firmicutes; c__Clostridia; o__Clostridiales; f__; g__; s__ |
| denovo140 | 0.064815242 | 0.004397183 | 0.03999831 | k__Bacteria; p__Proteobacteria; c__Alphaproteobacteria; o__RF32; f__; g__; s__ |
| denovo381 | 0.062381889 | 0.004437596 | 0.040261084 | k__Bacteria; p__Firmicutes; c__Clostridia; o__Clostridiales; f__Ruminococcaceae; g__; s__ |
| denovo329 | -0.062452566 | 0.004686317 | 0.041863229 | k__Bacteria; p__Firmicutes; c__Clostridia; o__Clostridiales; f__Lachnospiraceae; g__Dorea; s__ |
| denovo153 | -0.063046091 | 0.004817765 | 0.042699867 | k__Bacteria; p__Firmicutes; c__Clostridia; o__Clostridiales; f__Lachnospiraceae; g__Dorea; s__ |
| denovo9 | 0.0615694 | 0.00508475 | 0.044378959 | k__Bacteria; p__Firmicutes; c__Clostridia; o__Clostridiales; f__; g__; s__ |
| denovo258 | 0.060067263 | 0.005713174 | 0.048233113 | k__Bacteria; p__Firmicutes; c__Clostridia; o__Clostridiales; f__Ruminococcaceae; g__; s__ |
| denovo182 | -0.060991397 | 0.005847258 | 0.049039078 | k__Bacteria; p__Firmicutes; c__Clostridia; o__Clostridiales; f__Ruminococcaceae; g__Ruminococcus; s__ |
| denovo82 | 0.060954575 | 0.005894054 | 0.049319085 | k__Bacteria; p__Bacteroidetes; c__Bacteroidia; o__Bacteroidales; f__[Odoribacteraceae]; g__Odoribacter; s__ |
| denovo852 | -0.061692546 | 0.005899246 | 0.049350115 | k__Bacteria; p__Firmicutes; c__Clostridia; o__Clostridiales; f__Lachnospiraceae; g__Coprococcus; s__ |

**Additional file 1: Table S11.** FDR significant OTU associations with the MDS - collapsed to genus level. Number of significant associations = 6

| genus | Beta | Nom_P | FDR.P |
| --- | --- | --- | --- |
| k__Bacteria.p__Actinobacteria.c__Actinobacteria.o__Actinomycetales.f__Actinomycetaceae.g__Actinomyces | -0.100744127 | 4.68E-06 | 0.002939585 |
| k__Bacteria.p__Firmicutes.c__Clostridia.o__Clostridiales.f__Lachnospiraceae.g__Lachnospira | 0.100367069 | 4.90E-06 | 0.003031375 |
| k__Bacteria.p__Proteobacteria.c__Betaproteobacteria.o__Burkholderiales.f__Oxalobacteraceae.g__Oxalobacter | 0.095576056 | 1.41E-05 | 0.006937708 |
| k__Bacteria.p__Tenericutes.c__Mollicutes.o__Anaeroplasmatales.f__Anaeroplasmataceae.g__ | 0.093903161 | 1.95E-05 | 0.009469311 |
| k__Bacteria.p__Firmicutes.c__Clostridia.o__Clostridiales.f__Lachnospiraceae.g__.Ruminococcus. | -0.090476866 | 3.97E-05 | 0.020350187 |
| k__Bacteria.p__Proteobacteria.c__Alphaproteobacteria.o__RF32.f__.g__ | 0.09025249 | 4.20E-05 | 0.021715137 |

**Additional file 1: Table S12.** FDR significant OTU associations with the HFD-index. Number of significant associations = 13

| OTU | Beta | Nom_P | FDR.P | Assigned.Taxonomy |
| --- | --- | --- | --- | --- |
| denovo328 | -0.1139098 | 1.79E-07 | 0.000239665 | k__Bacteria; p__Firmicutes; c__Clostridia; o__Clostridiales; f__Ruminococcaceae; g__Ruminococcus; s__ |
| denovo1390 | -0.104805478 | 1.46E-06 | 0.001061569 | k__Bacteria; p__Firmicutes; c__Clostridia; o__Clostridiales; f__Ruminococcaceae; g__; s__ |
| denovo852 | -0.10060868 | 4.04E-06 | 0.002197867 | k__Bacteria; p__Firmicutes; c__Clostridia; o__Clostridiales; f__Lachnospiraceae; g__Coprococcus; s__ |
| denovo1100 | -0.092954579 | 2.07E-05 | 0.006545259 | k__Bacteria; p__Firmicutes; c__Clostridia; o__Clostridiales; f__; g__; s__ |
| denovo697 | -0.090785162 | 3.42E-05 | 0.008975559 | k__Bacteria; p__Firmicutes; c__Clostridia; o__Clostridiales; f__Lachnospiraceae; g__; s__ |
| denovo230 | -0.088010292 | 6.18E-05 | 0.012904336 | k__Bacteria; p__Firmicutes; c__Erysipelotrichi; o__Erysipelotrichales; f__Erysipelotrichaceae; g__cc_115; s__ |
| denovo237 | 0.083811619 | 0.000136645 | 0.020831782 | k__Bacteria; p__Firmicutes; c__Clostridia; o__Clostridiales; f__Lachnospiraceae; g__Blautia; s__producta |
| denovo765 | -0.082711788 | 0.00016345 | 0.02318581 | k__Bacteria; p__Firmicutes; c__Clostridia; o__Clostridiales; f__Ruminococcaceae; g__Ruminococcus; s__ |
| denovo728 | -0.081663729 | 0.00020343 | 0.026412316 | k__Bacteria; p__Firmicutes; c__Clostridia; o__Clostridiales; f__Ruminococcaceae; g__Anaerotruncus; s__ |
| denovo27 | -0.079062614 | 0.000292739 | 0.032763458 | k__Bacteria; p__Firmicutes; c__Clostridia; o__Clostridiales; f__Lachnospiraceae; g__[Ruminococcus]; s__gnavus |
| denovo383 | 0.078305417 | 0.000318344 | 0.03442331 | k__Bacteria; p__Firmicutes; c__Clostridia; o__Clostridiales; f__Lachnospiraceae; g__; s__ |
| denovo193 | -0.078153743 | 0.000362633 | 0.037163996 | k__Bacteria; p__Firmicutes; c__Erysipelotrichi; o__Erysipelotrichales; f__Erysipelotrichaceae; g__; s__ |
| denovo345 | -0.076103886 | 0.000476646 | 0.043617439 | k__Bacteria; p__Firmicutes; c__Clostridia; o__Clostridiales; f__Christensenellaceae; g__; s__ |

**Additional file 1: Table S13.** FDR significant OTU associations - collapsed to genus with the HFD-index. Number of significant associations = 1

| genus | Beta | Nom_P | FDR.P |
| --- | --- | --- | --- |
| k__Bacteria.p__Firmicutes.c__Erysipelotrichi.o__Erysipelotrichales.f__Erysipelotrichaceae.g__cc_115 | -0.099485224 | 5.40E-06 | 0.010604852 |

**Additional file 1: Table S14.** Results of mixed effects models with extracted PCs from ordination of unifrac distances representing the first 10 axis, against the Healthy Eating Index

| Axes | AIC | t.val | beta | P |
| --- | --- | --- | --- | --- |
| 1 | -3296.650386 | -4.496321524 | -0.096984864 | 7.29E-06 |
| 2 | -5528.711355 | -6.670707661 | -0.134857177 | 3.43E-11 |
| 3 | -5671.825213 | -0.991648102 | -0.020647374 | 0.321598692 |
| 4 | -6998.743985 | 3.945427338 | 0.081484075 | 8.29E-05 |
| 5 | -7111.594316 | -1.338721157 | -0.028441054 | 0.18086218 |
| 6 | -7451.968663 | -1.315787141 | -0.028644278 | 0.188387946 |
| 7 | -7596.835078 | -0.475970084 | -0.010380687 | 0.634208105 |
| 8 | -7869.306041 | -2.13425612 | -0.046501588 | 0.033071605 |
| 9 | -8038.19036 | -0.293997715 | -0.006423123 | 0.768884166 |
| 10 | -8249.483104 | -2.297157081 | -0.04914144 | 0.021746547 |

**Additional file 1: Table S15.** Results of mixed effects models with extracted PCs from ordination of unifrac distances representing the first 10 axis, against the Mediterranean Dietary Score

| Axis | AIC | t.val | beta | P |
| --- | --- | --- | --- | --- |
| 1 | -3284.150355 | -2.762383042 | -0.05997444 | 0.005785296 |
| 2 | -5520.788074 | -6.031997604 | -0.122633199 | 1.98E-09 |
| 3 | -5672.666225 | -1.350746972 | -0.028178909 | 0.176903665 |
| 4 | -6984.779746 | 1.235775765 | 0.025666475 | 0.216632385 |
| 5 | -7112.631353 | -1.682345413 | -0.035800331 | 0.092658905 |
| 6 | -7453.574344 | -1.829393633 | -0.039984851 | 0.067784919 |
| 7 | -7598.182771 | -1.25486948 | -0.027445 | 0.209613978 |
| 8 | -7864.994793 | 0.482000835 | 0.010542258 | 0.630916465 |
| 9 | -8038.655411 | 0.742760957 | 0.016277174 | 0.457750566 |
| 10 | -8250.542373 | -2.517444061 | -0.053958829 | 0.011903271 |

**Additional file 1: Table S16.** Results of mixed effects models with extracted PCs from ordination of unifrac distances representing the first 10 axis, against the Healthy Food Diversity index

|  | AIC | t.val | beta | P |
| --- | --- | --- | --- | --- |
| 1 | -3278.842253 | 1.520219507 | 0.032756761 | 0.128718754 |
| 2 | -5488.490876 | -1.924397561 | -0.039103159 | 0.054555244 |
| 3 | -5671.576013 | -0.856515109 | -0.017757127 | 0.391836877 |
| 4 | -6983.360592 | 0.327855127 | 0.00676767 | 0.743139406 |
| 5 | -7110.268481 | -0.68194539 | -0.014431274 | 0.495440062 |
| 6 | -7450.267879 | -0.171551052 | -0.003717117 | 0.863868527 |
| 7 | -7600.591627 | -1.99718228 | -0.043270686 | 0.045963101 |
| 8 | -7884.12149 | 4.411583298 | 0.095304499 | 1.08E-05 |
| 9 | -8038.243449 | 0.373435319 | 0.00811259 | 0.708850685 |
| 10 | -8244.327636 | 0.332646131 | 0.007090716 | 0.739594734 |

Additional file 1: Table S17 - Grouping and conversions of Food Frequency Questionnaire data to components required for the Healthy Eating Index (HEI). Conversions were all calculated in RStudio (RStudio Team, 2015) using the appropriate Composition of foods integrated database (CoFid) published by Public Health England (Finglas et al., 2015)

| ***Adequacy Components*** | | | | | | | | | | |
| --- | --- | --- | --- | --- | --- | --- | --- | --- | --- | --- |
| **Whole fruit** | | | | | | | | | | |
| **Points** | | 5 | | | | | | | | |
| **Min:** | | No whole fruit | | | | | | | | |
| **Max:** | | >=0.4 cups per 1000kcal | | | | | | | | |
| **Notes** | | Whole fruits only i.e. excluding fruit juice; fruit juice ; Gives frequency per week, per day calculation and scoring in R  Tomatoes considered a vegetable following HEI guidelines | | | | | | | | |
| **Conversions** | | Grams to cups per frequency ; Conversions (portion g/g per cup) = cups per portion | | | | | | | | |
| ***Variable portion description*** | | | ***CoFids code*** | ***Full Food Name*** | ***Proportion*** | ***Portion size (g)*** | ***G per cup*** | ***Conversion*** | ***Proportionate conversion*** | |
| Apples | Apples (1 fruit) | | 14-012 | Apples, eating, average, raw | 1 | 100 | 50 | 2 |  |  |
| Bananas | Bananas (1fruit) | | 14-045 | Bananas | 1 | 100 | 60 | 1.66667 |  |  |
| Grapefruit | Grapefruit (half) | | 14-292 | Grapefruit, raw | 1 | 80 | 72 | 1.1111 |  |  |
| Grapes | Grapes (handful) | | 14-109 | Grapes, average | 1 | 80 | 60.4 | 1.324503 |  |  |
| Melon | Melon (1 slice) | | 14-296 | Melon, watermelon | 0.25 | 200 | 64 | 3.125 | 0.78125 | 2.92969 |
| Melon | Melon (1 slice) | | 50-921 | Melon, Honeydew | 0.25 | 200 | 64 | 3.125 | 0.78125 |  |
| Melon | Melon (1 slice) | | 50-923 | Melon, Galia | 0.25 | 200 | 64 | 3.125 | 0.78125 |  |
| Melon | Melon (1 slice) | | 14-295 | Melon, Canteloupe-type | 0.25 | 150 | 64 | 2.34375 | 0.58594 |  |
| Oranges | Oranges, satsumas, mandarins (1 fruit) | | 14-298 | Oranges | 1 | 120 | 72 | 1.6667 |  |  |
| Peaches | Peaches, plums, apricots (1 fruit) | | 14-299 | Peaches, raw | 1 | 70 | 61.6 | 1.13637 |  |  |
| Pears | Pears (1 fruit) | | 14-190 | Pears, average, raw | 1 | 170 | 64 | 2.65625 |  |  |
| Strawberries | Strawberries, raspberries, other berries, kiwi fruit (one fruit or handful) | | 14-260 | Strawberries, raw | 1 | 100 | 61.6 | 1.62338 |  |  |
| TinnedFruit | Tinned fruit (handful) | | 14-096 | Fruit cocktail, canned in juice | 1 | 100 | 62.5 | 1.6 |  |  |
| DriedFruit | Dried fruit, e.g. raisins, prunes (heaped tablespoon) | | 14-087 | Dried mixed fruit | 1 | 30 | 50 | 0.6 |  |  |

| **Total Fruit** |  | | | | | | |
| --- | --- | --- | --- | --- | --- | --- | --- |
| **Points** | 5 | | | | | | |
| **Notes** | All fruit in Whole fruit component, plus below | | | | | | |
| **Min:** | No whole fruit | | | | | | |
| **Max:** | >=0.4cups per 1000kcal | | | | | | |
| **Conversions** | Grams to cups per frequency ; Conversions (portion g/g per cup) = cups per portion | | | | | | |
| ***Variable name*** | ***Variable portion description*** | ***CoFids code*** | ***Full Food Name*** | ***Proportion*** | ***Portion size (g)*** | ***G per cup*** | ***Conversion*** |
| PureFruitJuice | Pure fruit juice (100%) e.g. orange, apple juice (cup) | 17-195 | Fruit juice drink, ready to drink | 1 | 160 | 100 | 1.6 |
| Smoothies | Smoothies (cup) | 14-281 | Orange juice, freshly squeezed | 1 | 250 | 100 | 2.5 |

| **Whole grains** | |  |  |  |  |  |
| --- | --- | --- | --- | --- | --- | --- |
| **Points** | 10 |  |  |  |  |  |
| **Min:** | No whole grain |  |  |  |  |  |
| **Max:** | >=15oz per 1000kcal | |  |  |  |  |
| **Notes** | Brown bread included here even though FFQ group includes it within refined carbs | | | |  |  |
| **Conversions** | Gram to oz per frequency | |  |  |  |  |
|  | ***VarName*** | ***VarDescription_portion*** | ***CoFids code*** | ***Full Food Name*** | ***Proportion*** | ***Portion size (g)*** |
|  | BrownBread | Brown bread/rolls | 11-456 | Brown bread, average | 1 | 36 |
|  | BrownRice | Brown rice | 11-443 | Brown rice, boiled | 1 | 180 |
|  | Crispbread | Crispbread, e.g. Ryvita | 11-511 | Crispbread, rye | 1 | 10 |
|  | HighFibreCereal | High Fibre cereals e.g. Branflakes, All Bran, Fruit and Fibre | 11-485 | All-Bran | 0.33 | 40 |
|  | HighFibreCereal | High Fibre cereals e.g. Branflakes, All Bran, Fruit and Fibre | 11-493 | Fruit 'n Fibre | 0.33 | 40 |
|  | HighFibreCereal | High Fibre cereals e.g. Branflakes, All Bran, Fruit and Fibre | 11-486 | Bran Flakes | 0.33 | 40 |
|  | Muesli | Muesli | 11-494 | Muesli, swiss style | 1 | 60 |
|  | Porridge | Porridge, Readybreak, oats | 11-569 | Porridge, made with water | 1 | 160 |
|  | WholemealBread | Wholemeal & granary bread/rolls | 11-476 | Wholemeal bread, average | 1 | 36 |
|  | WholemealPasta | Wholemeal pasta | 11-455 | Spaghetti, wholemeal, boiled | 1 | 230 |

| **Dairy (and dairy alternatives)** | | | | | | | | | | | | | | | | |
| --- | --- | --- | --- | --- | --- | --- | --- | --- | --- | --- | --- | --- | --- | --- | --- | --- |
| **Points** | | 10 | | | | | | | | | | | | | | |
| **Min:** | | 0 | | | | | | | | | | | | | | |
| **Max:** | | >=1.3cup per 1000kcal | | | | | | | | | | | | | | |
| **Notes** | | Includes soy milks and non-butter spread as in HEI guidelines  Only non-fat fraction considered. Fat also removed from non-dairy milks (eg soy) products for continuity | | | | | | | | | | | | | | |
| **Conversions** | | Lean dairy in gs calculated by subtracting fat per 100g (as in Cofids databased) from portion and converting to cups; those in ml converted by specific gravity and multiplying by standard ml per cup | | | | | | | | | | | | | | |
| ***Variable*** | ***Variable Description*** | | ***CoFids code*** | ***Full Food Name*** | ***Proportion*** | ***Portion size_g*** | ***Solid_fat/g/100g*** | ***Fat/portion (g)*** | ***Dairy/ portion(g)*** | ***Dairy/portion (oz)*** | ***Specific gravity*** | ***Dairy/portion_(ml)*** | ***dairy/ portion (cup)*** | | ***Conversion*** | |
| DairyDesserts | Dairy desserts (small pot) e.g. chocolate mousse, cream caramels | | 12-398 | Chocolate dairy desserts | 1 | 125 | 10.7 | 13.375 | 111.625 | 39.37460 | 1.08 | 103.3564815 | 0.43686 | |  |  |
| DairyFFYog | Full fat or Greek yoghurt (small pot) | | 12-375 | Yogurt, whole milk, fruit | 1 | 125 | 3 | 3.75 | 121.25 | 42.769725 | 1.08 | 112.2685185 | 0.474530 | |  |  |
| DairyLFYog | Low fat yoghurt, fromage frais (small pot) | | 12-380 | Yogurt, low fat, fruit | 1 | 125 | 1.1 | 1.375 | 123.625 | 43.6074825 | 1.08 | 114.4675926 | 0.483825897 | |  |  |
| GoatsMilk | Goats' milk | | 12-328 | Goats milk, pasteurised | 1 | 585 | 3.7 | 21.645 | 563.355 | 198.7178427 | 1.08 | 521.625 | 2.204778469 | |  |  |
| EvaporatedMilk | Evaporated milk | | 12-033 | Evaporated milk, whole | 1 | 585 | 9.4 | 54.99 | 530.01 | 186.9557274 | 1.07 | 495.3364486 | 2.093663334 | |  |  |
| ChannelIslandMilk | Channel Islands milk | | 12-018 | Channel island milk, whole, pasteurised | 1 | 585 | 5.1 | 29.835 | 555.165 | 195.8289021 | 1.03 | 538.9951456 | 2.278197732 | |  |  |
| FullMilk | Full cream milk | | 12-315 | Whole milk, average | 1 | 585 | 3.9 | 22.815 | 562.185 | 198.3051369 | 1.03 | 545.8106796 | 2.30700529 | |  |  |
| RiceMilk | Rice milk | | LD01 | Rice milk | 1 | 585 | 0 | 0 | 585 | 206.3529 | 1.03 | 567.961165 | 2.400629854 | |  |  |
| SemiSkimmedMilk | Semi-skimmed milk | | 12-312 | Semi-skimmed milk, average | 1 | 585 | 1.7 | 9.945 | 575.055 | 202.8449007 | 1.03 | 558.3058252 | 2.359819147 | |  |  |
| SkimmedMilk | Skimmed milk | | 12-306 | Skimmed milk, average | 1 | 585 | 0.2 | 1.17 | 583.83 | 205.9401942 | 1.03 | 566.8252427 | 2.395828595 | |  |  |
| SoyaMilk | Soya milk | | 12-331 | Soya, non-dairy alternative to milk, unsweetened | 1 | 585 | 1.6 | 9.36 | 575.64 | 203.0512536 | 1.03 | 558.8737864 | 2.362219777 | |  |  |
| Dairysingle | Single or sour cream (tablespoon) | | 12-332 | Cream, fresh, single | 1 | 15 | 19.1 | 2.865 | 12.135 | 4.2804999 | 1 | 12.135 | 0.051291611 | |  |  |
| Dairydouble | Double or clotted cream (tablespoon) | | 12-334 | Cream, fresh, double, including Jersey cream | 1 | 30 | 53.7 | 16.11 | 13.89 | 4.8995586 | 0.94 | 14.77659574 | 0.062456976 | |  |  |
| CholLowerSpread | Cholesterol lowering fat spreads e.g. Benecol (teaspoon) | | 17-552 | Fat spread (40% fat), not polyunsaturated | 1 | 5 | 37.5 | 1.875 | 3.125 | 1.1023125 | 0.911 | 3.430296378 | 0.014499005 | |  |  |
| IceCream | Ice cream, choc ices | | 12-384 | Choc ice | 0.33 | 75 | 21.7 | 16.275 | 58.725 | 20.7146565 | 0.61 | 96.2704918 | 0.406911301 | | 0.1343 | 0.443658042 |
| IceCream | Ice cream, choc ices | | 12-387 | Ice cream, dairy, vanilla | 0.66 | 75 | 9.8 | 7.35 | 67.65 | 23.862861 | 0.61 | 110.9016393 | 0.468753504 | | 0.309377313 |  |
|  |  | |  |  |  |  |  |  |  |  | **density_g_per_ml** | **g_per_cup** | |  |  |  |
| Butter | Butter (teaspoon) | | 17-485 | Butter | 1 | 5 | 82.5 | 4.125 | 0.875 | 0.3086475 | 0.911 | 215.5318839 | | 0.004059724 |  |  |
| RedFatButter | Reduced fat butter (teaspoon) | | 17-016 | Blended spread, (40% fat) | 1 | 5 | 39.6 | 1.98 | 3.02 | 1.0652748 | 0.911 | 215.5318839 | | 0.014011848 |  |  |
| BlockMarg | Block margarine, e.g. Stork, Krona (teaspoon) | | 17-018 | Margarine, hard, animal and vegetable fats | 1 | 5 | 79.3 | 3.965 | 1.035 | 0.3650859 | 0.96 | 227.1247075 | | 0.004556968 |  |  |
| CoffeeWhitener | Coffee whitener, e.g. Coffee-mate (teaspoon) | | 12-027 | Coffeemate | 1 | 3 | 34.9 | 1.047 | 1.953 | 0.68890122 | 0.56 | 132.4894127 | | 0.0147408 |  |  |
| Dairycheese | Cheese, e.g. cheddar, brie, edam (matchbox size) | | 12-355 | Cheese, Edam | 0.1 | 40 | 26.0 | 10.4 | 29.6 | 10.441104 | 0.34 | 80.44000058 | | 0.367976129 | 0.036797613 | 0.332620584 |
| Dairycheese | Cheese, e.g. cheddar, brie, edam (matchbox size) | | 12-344 | Cheese, Brie | 0.1 | 40 | 29.1 | 11.64 | 28.36 | 10.0037064 | 0.34 | 80.44000058 | | 0.352560912 | 0.035256091 |  |
| Dairycheese | Cheese, e.g. cheddar, brie, edam (matchbox size) | | 12-359 | Cheese, hard, average | 0.8 | 40 | 34.5 | 13.8 | 26.2 | 9.241788 | 0.34 | 80.44000058 | | 0.3257086 | 0.26056688 |  |
| DairyCottageCheese | Cottage cheese, low fat soft cheese (2 tablespoons) | | 12-351 | Cheese, cottage, plain | 1 | 55 | 4.3 | 2.365 | 52.635 | 18.5664699 | 0.34 | 80.44000058 | | 0.654338633 |  |  |
| DairyLFcheese | Low fat cheese e.g. reduced fat cheddar (matchbox size) | | 12-348 | Cheese, cheddar type, half fat | 1 | 30 | 15.8 | 4.74 | 25.26 | 8.9102124 | 0.34 | 80.44000058 | | 0.314022872 |  |  |
| DriedMilk | Dried milk | | 12-030 | Dried skimmed milk | 1 | 50 | 0.6 | 0.3 | 49.7 | 17.531178 | 0.21 | 49.68352977 | | 1.000331503 |  |  |
| LowFatSpread | Low fat spread, e.g. Outline, Gold (teaspoon) | | 17-552 | Fat spread (20-25% fat), not polyunsaturated | 1 | 5 | 25.5 | 1.275 | 3.725 | 1.3139565 | 0.34 | 80.44000058 | | 0.046307807 |  |  |
| OliveOilSpread | Olive oil spread (teaspoon) | | 17-025 | Fat spread (60% fat), with olive oil | 1 | 5 | 62.70 | 3.135 | 1.865 | 0.6578601 | 0.34 | 80.44000058 | | 0.023184982 |  |  |
| OtherSoftMarg | Other soft margarine, dairy spreads, e.g. Blue Band, Clover (teaspoon) | | 17-020 | Margarine, soft, not polyunsaturated | 1 | 5 | 80 | 4 | 1 | 0.35274 | 0.34 | 80.44000058 | | 0.012431626 |  |  |
| PufaMarg | Polyunsaturated margarine, e.g. Flora, sunflower (teaspoon) | | 17-021 | Margarine, soft, polyunsaturated | 1 | 5 | 82.8 | 4.14 | 0.86 | 0.3033564 | 0.34 | 80.44000058 | | 0.010691198 |  |  |
| VLowFatSpread | Very low fat spread (teaspoon) e.g. Diet Flora | | 17-554 | Fat spread (5% fat) | 1 | 5 | 5 | 0.25 | 4.75 | 1.675515 | 0.34 | 80.44000058 | | 0.059050223 |  |  |

| **Total Protein Foods** | | | | | |  |  | | |  | |  |  | | |  | |  |
| --- | --- | --- | --- | --- | --- | --- | --- | --- | --- | --- | --- | --- | --- | --- | --- | --- | --- | --- |
| **Points** | | 5 |  | | |  |  | | |  | |  |  | | |  | |  |
| **Min:** | | 0 |  | | |  |  | | |  | |  |  | | |  | |  |
| **Max:** | | >=2.5 oz per 1000kcal | | | |  |  | | |  | |  |  | | |  | |  |
| **Notes** | | **Includes Seafood and plant protein (**below), and protein from peas and beans considered up to where the max protein standard is met, and then included in greens/total vegetables | | | | | | | | | | | | | | | | |
| **Conversions** | | g to oz ; protein from beans peas oz calculated and added to total, if total did not already exceed 2.5oz (Calculation to add back in to greens and beans below) | | | | | | | | | | | | | | | |  |
| ***VarName*** | ***VarDescription*** | | | ***CoFids code*** | ***Full Food Name*** | | | ***Proportion*** | ***Portion size*** | | ***Protein_per_100g*** | | | ***Prot_per_portion*** | ***Proportionate conversion*** | | | |
| Eggs | Eggs as boiled, fried, scrambled, etc. (one) | | | 12-806 | Eggs, chicken, boiled | | | 1 | 50 | | 14.1 | | | 7.05 |  | |  | |
| Lasagne | Lasagne, moussaka | | | 19-346 | Lasagne, homemade | | | 1 | 420 | | 9.6 | | | 40.32 |  | |  | |
| MFBacon | Bacon or gammon | | | 19-003 | Bacon rashers, back, grilled | | | 1 | 25 | | 23.2 | | | 5.8 |  | |  | |
| MFBeef | Beef: roast, steak, mince, stew or casserole | | | 18-081 | Beef, stewing steak, stewed, lean and fat | | | 0.01 | 140 | | 29.2 | | | 40.88 | 0.292 | | 26.912 | |
| MFBeef | Beef: roast, steak, mince, stew or casserole | | | 18-091 | Beef, topside, roasted well-done, lean and fat | | | 0.26 | 90 | | 32.8 | | | 29.52 | 8.528 | |  | |
| MFBeef | Beef: roast, steak, mince, stew or casserole | | | 18-472 | Beef, rump steak, lean and fat, fried | | | 0.33 | 144 | | 28.4 | | | 40.896 | 9.372 | |  | |
| MFBeef | Beef: roast, steak, mince, stew or casserole | | | 18-470 | Beef, mince, stewed | | | 0.4 | 140 | | 21.8 | | | 30.52 | 8.72 | |  | |
| MFburgers | Beefburgers | | | 19-030 | Beefburgers, chilled/frozen, grilled | | | 1 | 78 | | 26.5 | | | 20.67 |  | |  | |
| MFCornedBeef | Corned Beef, Spam, luncheon meats | | | 19-135 | Luncheon meat, canned | | | 0.5 | 14 | | 12.9 | | | 1.806 | 6.45 | | 19.4 | |
| MFCornedBeef | Corned Beef, Spam, luncheon meats | | | 19-128 | Corned beef, canned | | | 0.5 | 38 | | 25.9 | | | 9.842 | 12.95 | |  | |
| MFHam | Ham, cured meats & chorizo | | | 19-308 | Ham | | | 1 | 23 | | 18.4 | | | 4.232 |  | |  | |
| MFLamb | Lamb: roast, chops or stew | | | 18-187 | Lamb, stewing, stewed, lean and fat | | | 0.16 | 140 | | 24.4 | | | 34.16 | 3.904 | | 27.076 | |
| MFLamb | Lamb: roast, chops or stew | | | 18-477 | Lamb, loin chops, lean and fat, grilled | | | 0.27 | 70 | | 26.5 | | | 18.55 | 7.155 | |  | |
| MFLamb | Lamb: roast, chops or stew | | | 18-480 | Lamb, leg, whole, lean and fat, roast, medium | | | 0.57 | 90 | | 28.1 | | | 25.29 | 16.017 | |  | |
| MFLiver | Liver, liver pate, liver sausage | | | 18-494 | Liver, lamb, fried | | | 0.33 | 100 | | 30.1 | | | 30.1 | 9.933 | | 18.249 | |
| MFLiver | Liver, liver pate, liver sausage | | | 19-317 | Pate, liver | | | 0.66 | 80 | | 12.6 | | | 10.08 | 8.316 | |  | |
| MFPies | Savoury pies, e.g. meat pie, pork pie, pasties, steak & kidney pie, sausage rolls | | | 19-066 | Sausage rolls, puff pastry | | | 0.25 | 60 | | 9.9 | | | 5.94 | 2.475 | | 9.05 | |
| MFPies | Savoury pies, e.g. meat pie, pork pie, pasties, steak & kidney pie, sausage rolls | | | 19-316 | Cornish pastie, retail | | | 0.25 | 155 | | 6.7 | | | 10.385 | 1.675 | |  | |
| MFPies | Savoury pies, e.g. meat pie, pork pie, pasties, steak & kidney pie, sausage rolls | | | 19-069 | Steak and kidney/Beef pie, individual, chilled/frozen, baked | | | 0.25 | 160 | | 8.8 | | | 14.08 | 2.2 | |  | |
| MFPies | Savoury pies, e.g. meat pie, pork pie, pasties, steak & kidney pie, sausage rolls | | | 19-063 | Pork pie, individual | | | 0.25 | 140 | | 10.8 | | | 15.12 | 2.7 | |  | |
| MFPork | Pork: roast, chops or stew | | | 18-482 | Pork, diced, casseroled, lean only | | | 0.07 | 140 | | 31.7 | | | 44.38 | 2.219 | | 30.082 | |
| MFPork | Pork: roast, chops or stew | | | 18-252 | Pork, loin chops, grilled, lean and fat | | | 0.46 | 75 | | 29.0 | | | 21.75 | 13.34 | |  | |
| MFPork | Pork: roast, chops or stew | | | 18-485 | Pork, leg joint, lean and fat, roast, medium | | | 0.47 | 90 | | 30.9 | | | 27.81 | 14.523 | |  | |
| MFPoultry | Chicken or other poultry e.g. turkey | | | 18-361 | Turkey, meat, average, roasted | | | 0.17 | 90 | | 31.2 | | | 28.08 | 5.304 | | 27.963 | |
| MFPoultry | Chicken or other poultry e.g. turkey | | | 18-331 | Chicken, meat, average, roasted | | | 0.83 | 100 | | 27.3 | | | 27.3 | 22.659 | |  | |
| MFSausages | Sausages | | | 19-077 | Beef sausages, chilled, grilled | | | 0.14 | 40 | | 13.3 | | | 5.32 | 1.862 | | 14.332 | |
| MFSausages | Sausages | | | 19-080 | Pork sausages, chilled, grilled | | | 0.86 | 40 | | 14.5 | | | 5.8 | 12.47 | |  | |

| **Seafood and plant protein** | | | | | |  |  | | |  | |  | |  | |  | |  |
| --- | --- | --- | --- | --- | --- | --- | --- | --- | --- | --- | --- | --- | --- | --- | --- | --- | --- | --- |
| **Points** | 5 | |  | | |  |  | | |  | |  | |  | |  | |  |
| **Min:** | 0 | |  | | |  |  | | |  | |  | |  | |  | |  |
| **Max:** | >= 0.8 oz per 1000kcal | | | | |  |  | | |  | |  | |  | |  | |  |
| **Notes** | Protein from peas and beans used to meet total protein food standard added | | | | | | | | |  | |  | |  | |  | |  |
| **Conversions** | g to oz ; protein from beans peas oz calculated and added to total, if total did not already exceed 2.5oz (Calculation to add back in to greens and beans below) | | | | | | | | | | | | | | | | |  |
| **Source of sub-component** | **VarName** | | **VarDescription** | | | **CoFids code** | **Full Food Name** | | | **Proportion** | | **Portion size** | | **Protein_g_per_100g** | | **Protein_per_portion** | | **Proportionate conversion** |
| MFFishFingers | | Fish fingers, fish cakes & breaded fish | | 16-281 | Fish cakes, grilled | | | 0.33 | 50 | | 9.9 | | 4.95 | | 1.6335 | | 3.3363 | |
| MFFishFingers | | Fish fingers, fish cakes & breaded fish | | 16-288 | Fish fingers, cod, grilled | | | 0.66 | 60 | | 4.3 | | 2.58 | | 1.7028 | |  | |
| MFFishroe | | Fish roe, taramasalata | | 16-307 | Taramasalata | | | 1 | 45 | | 3.2 | | 1.44 | |  | |  | |
| MFFriedfish | | Fried fish in batter, as in fish and chips | | 16-110 | Plaice, in batter, fried in blended oil | | | 0.06 | 200 | | 15.2 | | 30.4 | | 1.824 | | 26.9244 | |
| MFFriedfish | | Fried fish in batter, as in fish and chips | | 16-063 | Haddock, coated in crumbs, frozen, fried in blended oil | | | 0.24 | 170 | | 11.8 | | 20.06 | | 4.8144 | |  | |
| MFFriedfish | | Fried fish in batter, as in fish and chips | | 16-021 | Cod, in batter, fried in blended oil | | | 0.7 | 180 | | 16.1 | | 28.98 | | 20.286 | |  | |
| MFOilyfish | | Oily fish, fresh or canned, e.g. tuna, mackerel, kippers, salmon, sardines, herring | | 16-196 | Mackerel, smoked | | | 0.06 | 150 | | 18.9 | | 28.35 | | 1.701 | | 20.50324 | |
| MFOilyfish | | Oily fish, fresh or canned, e.g. tuna, mackerel, kippers, salmon, sardines, herring | | 16-328 | Sardines, canned in brine, drained | | | 0.08 | 100 | | 21.5 | | 21.5 | | 1.72 | |  | |
| MFOilyfish | | Oily fish, fresh or canned, e.g. tuna, mackerel, kippers, salmon, sardines, herring | | 16-207 | Salmon, smoked | | | 0.16 | 56 | | 25.4 | | 14.224 | | 2.27584 | |  | |
| MFOilyfish | | Oily fish, fresh or canned, e.g. tuna, mackerel, kippers, salmon, sardines, herring | | 16-327 | Salmon, grilled | | | 0.18 | 100 | | 19.8 | | 19.8 | | 3.564 | |  | |
| MFOilyfish | | Oily fish, fresh or canned, e.g. tuna, mackerel, kippers, salmon, sardines, herring | | 16-339 | Tuna, canned in brine, drained | | | 0.52 | 92 | | 23.5 | | 21.62 | | 11.2424 | |  | |
| MFShellfish | | Shellfish, e.g. crab, prawns, mussels | | 16-239 | Prawns, boiled | | | 1 | 60 | | 22.6 | | 13.56 | |  | |  | |
| MFWhitefish | | Other white fish, fresh or frozen, e.g. cod, plaice, sole, haddock, halibut | | 16-108 | Plaice, frozen, steamed | | | 0.06 | 130 | | 14.1 | | 18.33 | | 1.0998 | | 22.743 | |
| MFWhitefish | | Other white fish, fresh or frozen, e.g. cod, plaice, sole, haddock, halibut | | 16-049 | Haddock, steamed | | | 0.24 | 120 | | 20.9 | | 25.08 | | 6.0192 | |  | |
| MFWhitefish | | Other white fish, fresh or frozen, e.g. cod, plaice, sole, haddock, halibut | | 16-017 | Cod, steamed | | | 0.7 | 120 | | 18.6 | | 22.32 | | 15.624 | |  | |
| NutsSalted | | Salted nuts e.g. peanuts, cashews (handful) | | 14-812 | Cashew nuts, roasted and salted | | | 0.2 | 25 | | 20.5 | | 5.125 | | 1.025 | | 5.965 | |
| NutsSalted | | Salted nuts e.g. peanuts, cashews (handful) | | 14-834 | Peanuts, roasted and salted | | | 0.8 | 25 | | 24.7 | | 6.175 | | 4.94 | |  | |
| NutsUnsalted | | Unsalted nuts, e.g. brazil, walnuts (handful) | | 14-871 | Brazil nuts | | | 0.4 | 10 | | 14.3 | | 1.43 | | 0.572 | | 2.336 | |
| NutsUnsalted | | Unsalted nuts, e.g. brazil, walnuts (handful) | | 14-879 | Walnuts | | | 0.6 | 20 | | 14.7 | | 2.94 | | 1.764 | |  | |
| PeanutButter | | Peanut butter (teaspoon) | | 14-876 | Peanut butter, smooth | | | 1 | 20 | | 22.8 | | 4.56 | |  | |  | |
| Seeds | | Seeds e.g. Sunflower, pumpkin (tablespoon) | | 14-845 | Sunflower seeds | | | 0.5 | 16 | | 20.5 | | 3.28 | | 1.64 | | 3.592 | |
| Seeds | | Seeds e.g. Sunflower, pumpkin (tablespoon) | | 14-842 | Pumpkin seeds | | | 0.5 | 16 | | 24.4 | | 3.904 | | 1.952 | |  | |
| Tofu | | Meat substitutes e.g. tofu, soyameat, textured vegetable protein, vegeburger | | 50-723 | Tofu, soya bean, steamed | | | 0.4 | 120 | | 8.1 | | 9.72 | | 3.888 | | 9.4656 | |
| Tofu | | Meat substitutes e.g. tofu, soyameat, textured vegetable protein, vegeburger | | 15-331 | Vegeburger, retail, grilled | | | 0.6 | 56 | | 16.6 | | 9.296 | | 5.5776 | |  | |

| **Greens and Beans** | | |  | |  | |  | | |  |  | |  | | |
| --- | --- | --- | --- | --- | --- | --- | --- | --- | --- | --- | --- | --- | --- | --- | --- |
| **Points** | | 5 |  | |  | |  | | |  |  | |  | | |
| **Min:** | | 0 |  | |  | |  | | |  |  | |  | | |
| **Max:** | | >=0.2 cups / 1000kcal | | |  | |  | | |  |  | |  | | |
| **Notes** | Highlighted Beans and Peas used for protein calculations in total protein and Seafood and plant protein; amount of beans and peas considered is that minus the amount required to meet protein standard; Foods considered to be falling within this category ascertained from USDA guidelines | | | | | | | | | | | | | | |
| **Conversion** | | Frequency to cups; Amount of peas and beans previously used to meet protein standards removed first. Calculation 2.5oz | | | | | | | | | | |  | | |
|  | | | | | | | | | | | | | | | |
| **VarName** | | **VarDescription** | | **CoFids code** | | **Full Food Name** | | **Proportion** | **Portion size** | | | **Protein_g_per_100** | | **Protein_per_portion** | **Portion_cups** |
| BakedBeans | | Baked beans | | 13-044 | | Baked beans, canned in tomato sauce, re-heated | | 1 | 135 | | | 5.2 | | 7.02 | 0.53 |
| Beansprouts | | Beansprouts | | 13-426 | | Beansprouts, mung, raw | | 1 | 20 | | | 2.9 | | 0.58 | 0.53 |
| DriedLentils | | Pulses e.g. lentils, beans, peas | | 13-434 | | Lentils, red, split, dried, boiled in unsalted water | | 1 | 70 | | | 7.6 | | 5.32 | 0.27 |
| GreenBeans | | Green beans, broad beans, runner beans | | 13-432 | | Green beans/French beans, frozen, boiled in unsalted water | | 1 | 90 | | | 1.8 | | 1.62 | 0.82 |
| Peas | | Peas | | 13-440 | | Peas, frozen, boiled in unsalted water | | 1 | 70 | | | 6 | | 4.2 | 0.49 |

| ***Green Vegetables*** |  | |  | |  |  |  |
| --- | --- | --- | --- | --- | --- | --- | --- |
| **VarName** | **VarDescription** | **Food Code (5th)** | | **Full Food Name** | **Proportion** | **Portion size** | **Portion_cups** |
| Broccoli | Broccoli, spring green, kale | 13-172 | | Broccoli, green, boiled in unsalted water | 1 | 85 | 0.55 |
| GreenSalad | Green salad, lettuce, cucumber, celery | 15-380 | | Salad, green | 1 | 30 | 0.8 |
| Spinach | Spinach | 13-457 | | Spinach, boiled in unsalted water | 1 | 90 | 0.47 |
| Watercress | Watercress | 13-462 | | Watercress, raw | 1 | 20 | 0.59 |

| **Total Vegetables** | | | | |  | |  | | |  |  | |  | |
| --- | --- | --- | --- | --- | --- | --- | --- | --- | --- | --- | --- | --- | --- | --- |
| **Points** | | 5 |  | |  | |  | | |  |  | |  | |
| **Min:** | | 0 |  | |  | |  | | |  |  | |  | |
| **Max:** | | >=0.825 cups per 1000kcal | | | | |  | | |  |  | |  | |
| **Notes** | | Greens and beans included - potatoes included | | | | |  | | |  |  | |  | |
| **Conversion** | | g to cups per frequency | | | | |  | | |  |  | |  | |
| **VarName** | **VarDescription** | | | **Food Code (5th)** | | **Full Food Name** | | **Proportion** | **Portion size** | | | **Cups** | | **Proportionate conversion** |
| Avocado | Avocado | | | 14-037 | | Avocado, average | | 1 | 75 | | | 0.5 | |  |
| Beetroot | Beetroot | | | 13-166 | | Beetroot, pickled, drained | | 1 | 40 | | | 0.23 | |  |
| BrusselsSprouts | Brussel sprouts | | | 13-179 | | Brussels sprouts, boiled in unsalted water | | 1 | 90 | | | 0.58 | |  |
| Cabbage | Cabbage | | | 13-444 | | Cabbage, boiled in unsalted water, average | | 1 | 95 | | | 0.63 | |  |
| Carrots | Carrots | | | 13-447 | | Carrots, old, boiled in unsalted water | | 1 | 60 | | | 0.39 | |  |
| Cauliflower | Cauliflower | | | 13-217 | | Cauliflower, boiled in unsalted water | | 1 | 90 | | | 0.73 | |  |
| Coleslaw | Coleslaw | | | 15-077 | | Coleslaw, with mayonnaise, retail | | 1 | 45 | | | 0.38 | |  |
| Garlic | Garlic (clove) | | | 13-244 | | Garlic, raw | | 1 | 5 | | | 0.04 | |  |
| Marrow | Marrow, courgettes | | | 13-231 | | Courgette, boiled in unsalted water | | 1 | 90 | | | 0.4 | |  |
| Mushrooms | Mushrooms | | | 13-286 | | Mushrooms, common, fried in corn oil | | 1 | 56 | | | 0.52 | |  |
| Onions | Onions | | | 13-307 | | Onions, fried in corn oil | | 1 | 60 | | | 0.29 | |  |
| Parsnips | Parsnips, turnips, swedes | | | 13-454 | | Parsnip, boiled in unsalted water | | 0.33 | 65 | | | 0.42 | | 0.1386 |
| Parsnips | Parsnips, turnips, swedes | | | 50-834 | | Turnip, boiled in unsalted water | | 0.33 | 60 | | | 0.39 | | 0.1287 |
| Parsnips | Parsnips, turnips, swedes | | | 50-820 | | Swede, boiled in unsalted water | | 0.33 | 60 | | | 0.25 | | 0.0825 |
| Sweetcorn | Sweetcorn | | | 13-459 | | Sweetcorn, kernels, canned, re-heated, drained | | 1 | 85 | | | 0.52 | |  |
| SweetPeppers | Sweet peppers | | | 13-318 | | Peppers, capsicum, green, raw | | 1 | 80 | | | 0.5369 | |  |
| Tomatoes | Tomatoes | | | 13-460 | | Tomatoes, raw | | 1 | 85 | | | 0.47 | |  |
| Watercress | Watercress | | | 13-462 | | Watercress, raw | | 1 | 20 | | | 0.59 | |  |
| Boiledpotato | Boiled, mashed, instant or one jacket potato | | | 13-421 | | Old potatoes, boiled in unsalted water | | 1 | 180 | | | 0.2 | |  |

| **Fatty Acids** | |  |
| --- | --- | --- |
| **Points** | 10 |  |
| **Min:** | < 1.2 (ratio) |  |
| **Max:** | >= 2.5 (ratio) |  |
| **Notes** |  |  |
| **Conversion** | Calculated as the ratio of mono + polyunsat Fas / sat fas | |
|  | VarName | VarDescription |
|  | mono_fa_g | Mono - unsaturated fatty acids |
|  | poly_fa_g | Poly-unsaturated fatty acids |
|  | satd_fat_g | Saturated fatty acids |

| **Moderation Components** | | | | | | | | |
| --- | --- | --- | --- | --- | --- | --- | --- | --- |
| **Points** | | 10 | | | | | | |
| **Min:** | | >=4.3 oz / 1000kcal | | | | | | |
| **Max:** | | <1.8 oz / 1000kcal | | | | | | |
| **Conversions** | | Frequency to oz | | | | | | |
| **VarName** | **VarDescription** | | **Food Code (5th)** | **Full Food Name** | **Proportion** | **Portion size** | **Portion.oz** | **Proportionate conversion** |
| BreakfastCereal | Breakfast cereal e.g. Cornflakes, Rice Krispies | | 11-490 | Corn Flakes | 0.5 | 30 | 10.5822 |  |
| BreakfastCereal | Breakfast cereal e.g. Cornflakes, Rice Krispies | | 11-497 | Rice Krispies | 0.5 | 30 | 10.5822 |  |
| Frosties | Sugar topped cereals e.g. Frosties | | 11-492 | Frosties | 1 | 40 | 14.1096 |  |
| NaanPoppdmTort | Naan, poppadoms, flour tortillas | | 11-463 | Naan bread | 0.33 | 13 | 4.58562 | 26.33792 |
| NaanPoppdmTort | Naan, poppadoms, flour tortillas | | 11-464 | Pappadums, takeaway | 0.33 | 51 | 17.98974 |  |
| NaanPoppdmTort | Naan, poppadoms, flour tortillas | | 11-632 | Tortillas, made with wheat flour | 0.33 | 160 | 56.4384 |  |
| WhiteBread | White bread/rolls | | 11-468 | White bread, sliced | 1 | 36 | 12.69864 |  |
| Whitepasta | White or green pasta, e.g. spaghetti, macaroni, noodles | | 11-448 | Macaroni, boiled | 1 | 230 | 81.1302 |  |
| WhiteRice | White rice | | 11-446 | White rice, easy cook, boiled | 1 | 180 | 63.4932 |  |

| **Sodium** |  |
| --- | --- |
| **Points** | 10 |
| **Min:** | >= 2g |
| **Max:** | <1.1 |
| **Notes** |  |
| **Conversions** | mg to g |

| **Empty Calories (Added sugar & Fats, Alcohol over daily limit)** | |
| --- | --- |
| **Points** | 20 |
| **Min:** | >50% of energy |
| **Max:** | <= 19 of energy |
| **Notes** | Sugar and Fat content of components already considered for their lean content only, and remaining components with little nutritional value; this component is the most problematic in terms of its working out - eg considers all fats bad? |
| **Conversions** | Considerations made for gender in terms of alcohol limit; calories for sugar and fat within food calculated by subtracted sugars from fruits and vegetables (natural sugars) from total sugars for all FFQ foods as in the Cofids database. Solid fat calculated as total solid fat within all foods listed within FFQ. |

**Additional file 1: Table S18**. Food frequency questionnaire foods assigned to each food grouping necessary to calculate the adjusted Mediterranean diet score following methods from Trichopoulou et al., 2005.

| **MDS component** | **FFQ items assigned to component** | **Score if above median intake** |
| --- | --- | --- |
| Vegetables | Spinach, Broccoli, GreenSalad, Watercress, Carrots, BrusselsSprouts, Cabbage, Marrow, Cauliflower, Parsnips, Leeks, Onions, Garlic, Mushrooms, SweetPeppers, Tomatoes, Sweetcorn, Beetroot, Coleslaw, Avocado, Boiledpotato | 1 |
| Legumes | Peas, GreenBeans, BakedBeans, Beansprouts, DriedLentils | 1 |
| Fruits and Nuts | Apples, Pears, Oranges, Grapefruit, Bananas, Grapes, Melon, Peaches, Strawberries, TinnedFruit, DriedFruit, PureFruitJuice, Smoothies, PeanutButter, NutsSalted, NutsUnsalted, Seeds | 1 |
| Cereal | WhiteBread, NaanPoppdmTort, BreakfastCereal, Frosties, WhiteRice, Whitepasta,  BrownBread, WholemealBread, Crispbread, Porridge, Muesli, HighFibreCereal,  BrownRice, WholemealPasta | 1 |
| Fish | MFFriedfish, MFFishFingers, MFWhitefish, MFOilyfish, MFShellfish, MFFishroe) | 1 |
| Meat | MFBeef, MFburgers, MFPork, MFLamb, MFPoultry, MFBacon, MFHam, MFCornedBeef, MFSausages, MFPies, MFLiver | 0 |
| Dairy | Dairysingle, Dairydouble, DairyLFYog, DairyFFYog, DairyDesserts, Dairycheese, DairyLFcheese,  DairyCottageCheese, Butter, RedFatButter, BlockMarg, PufaMarg, OliveOilSpread, OtherSoftMarg, LowFatSpread, VLowFatSpread, CholLowerSpread, IceCream, CoffeeWhitener, FullMilk, SkimmedMilk, DriedMilk, SemiSkimmedMilk, ChannelIslandMilk) | 0 |
| Unsaturated fat: Saturated fat ratio | N/A - total saturated, mono- & poly-saturated fat calcualted from FFQ data | 1 |
| Alcohol | Beer, Liqueurs, RedWine, WhiteWine, Spirits | (If below or above moderate range as stated in Trichopoulou methodology) 0 |

**Additional file 1: Table S19.** Food frequency questionnaire items assigned to each health value group. Consumption in grammes was multiplied by the health value. Score was then divided by the total possible (0.26) and multiplied by simpson index score to calculate the Healthy Food Diversity index (HFD-index). Heath value group and multiplier as in Drescher et al 2007.

| Health Value group | Food frequency food items | Health value mulitplied by |
| --- | --- | --- |
| Vegetables, fruits, leaf salades, juices | Apples, Avocado, Bananas, Beansprouts, Beetroot, Broccoli, BrusselsSprouts, Cabbage, Carrots, Cauliflower, Coleslaw, DriedFruit, DriedLentils, Garlic, Grapefruit, Grapes, GreenBeans, GreenSalad, Leeks, Marrow, Melon, Mushrooms, Onions, Oranges, Parsnips, Peaches, Pears, Peas, PureFruitJuice, Spinach, Strawberries, SweetPeppers, Sweetcorn, TinnedFruit, Tomatoes, Watercress, Smoothies | 0.2328 |
| Wholemeal | BrownRice, Crispbread, Muesli, Porridge, WholemealBread,  [WholemealPasta, HighFibreCereal | 0.2044 |
| Potatoes | Boiledpotato, PotatoSalad, ChipsRoastPots | 0.146 |
| White-meal products/peeled rice | BrownBread, Frosties, WhiteBread, WhiteRice, Whitepasta , NaanPoppdmTort, BreakfastCereal | 0.0876 |
| Snacks and sweets | Biscuitschoc, Biscuitsplain, Bunshome, Bunsready, Cakeshome, Cakesready, Chocolatebar, CreamCrackers, Crisps, Dietfizzy, FizzySoftDrinks, Fruitpiehome, Fruitpiesready, MilkPuddings, SpongePudhome, SpongePudready, SugarAdded, SweetsToffees, BiscuitsRedFat, CerealBars, ChocsMilk, ChocsDark | 0.0292 |
| Fish/low-fat meat/low-fat meat products | MFFishroe, MFOilyfish, MFPoultry, MFShellfish, MFWhitefish | 0.09 |
| Low-fat milk/low-fat dairy products | DairyCottageCheese, DairyLFYog, DriedMilk, GoatsMilk,LowFatSpread, RiceMilk, SemiSkimmedMilk, SoyaMilk, VLowFatSpread, DairyLFcheese |  |
| Milk/dairy products | ChannelIslandMilk, CoffeeWhitener, DairyDesserts, DairyFFYog, Dairycheese, Dairydouble, Dairysingle, EvaporatedMilk, FullMilk, IceCream | 0.05 |
| Meat products, sausages, eggs | Eggs, Lasagne, MFBacon, MFBeef, MFCornedBeef, MFFishFingers, MFFriedfish, MFHam, MFLamb, MFLiver, MFPork, MFSausages, MFburgers | 0.030 |
| Bacon | Bacon | 0.01 |
| Oilseed rape/walnut oil | N/A |  |
| Wheat germ oil/soybean oil | N/A |  |
| Corn oil/sunflower oil | FrenchDressing, Lowcalsaladcream, Mayo Otherdressing | 0.004 |
| Margarines/butter | BlockMarg, Butter, OtherSoftMarg, RedFatButter, OliveOilSpread, CholLowerSpread | 0.0024 |
